# An 80-million-year sulphur isotope record of pyrite burial over the Permian–Triassic

**DOI:** 10.1038/s41598-022-21542-4

**Published:** 2022-10-17

**Authors:** Jack Salisbury, Darren R. Gröcke, H. D. R. Ashleigh Cheung, Lee R. Kump, Tom McKie, Alastair Ruffell

**Affiliations:** 1 Department of Earth Sciences, Durham University, South Road, Durham, DH1 3LE UK; 2 College of Earth and Mineral Sciences, Pennsylvania State University, University Park, PA 16802 USA; 3 Shell UK Exploration and Production, 1 Altens Farm Road, Nigg, Aberdeen, AB12 3FY UK; 4 School of Natural and Built Environment (Elmwood Building), Queen’s University, Belfast, BT7 1NN UK

**Keywords:** Element cycles, Geochemistry, Geology, Sedimentology

## Abstract

Despite the extensive use of sulphur isotope ratios (δ^34^S) for understanding ancient biogeochemical cycles, many studies focus on specific time-points of interest, such as the end-Permian mass extinction (EPME). We have generated an 80 million-year Permian–Triassic δ^34^S_evap_ curve from the Staithes S-20 borehole, Yorkshire, England. The Staithes δ^34^S_evap_ record replicates the major features of the global curve, while confirming a new excursion at the Olenekian/Anisian boundary at ~ 247 million years ago. We incorporate the resultant δ^34^S_evap_ curve into a sulphur isotope box model. Our modelling approach reveals three significant pyrite burial events (i.e. PBEs) in the Triassic. In particular, it predicts a significant biogeochemical response across the EPME, resulting in a substantial increase in pyrite burial, possibly driven by Siberian Traps volcanism. Our model suggests that after ~ 10 million years pyrite burial achieves relative long-term stability until the latest Triassic.

The Permian–Triassic interval has attracted much attention due to significant biological and geochemical events, including the end-Permian mass extinction (EPME)—the most catastrophic extinction event of the Phanerozoic^1^. The EPME is associated with a reduction in marine species biodiversity on the order of 80–90%^2^, extinction amongst tetrapods, and a possible dieback of terrestrial vegetation^3^. Driven by volcanism from the Siberian Traps^4^, the EPME is intimately linked with increased CO_2_, CH_4_ and SO_2_ fluxes^5–7^, heightened global atmospheric and sea surface temperatures (SST)^8^, intensified chemical weathering^9^, ozone depletion^10^, a reduction in marine pH^11^ and an expansion of anoxic, and possibly euxinic, oceanic water masses^12,13^. It has been proposed that the Early Triassic represents a period of climatic, geochemical, and biological instability, delaying the recovery from the EPME^14–17^. Multiple SST changes^8,18^ likely coincided with major fluctuations in ocean chemistry expressed as excursions in the carbon and sulphur isotope geochemistry of marine carbonates and evaporites^14,16,17,19^, followed by conditions of relative stability in the Middle Triassic^15^.

Despite the biogeochemical significance of the Triassic, robust sulphur isotope data are sparse, with most studies focusing on specific, short periods of time, such as the EPME^20^ and the Smithian/Spathian boundary^14,17^. These records lack temporal coverage and fail to capture long-term biogeochemical conditions for the Triassic at high resolutions. One exception is by Song et al.^15^, who compiled a δ^34^S record of carbonate-associated sulphate (CAS) from the late Permian to Middle Triassic from sections in south China. However, CAS is prone to diagenetic alteration^21,22^, with much of the isotopic heterogeneity of δ^34^S_CAS_ records across the EPME attributed to post-depositional alteration^19,23^. Bernasconi et al.^19^ compiled a δ^34^S_evap_ record of sedimentary evaporites from the late Permian to Middle Triassic, including multiple sections across several countries in Europe. Although evaporites are less prone to diagenetic alteration^19^, their coverage in the sedimentary record is often sparse and not continuous, thus resulting in a lack of high-resolution δ^34^S_evap_ curves.

## Constructing a high-resolution δ^34^S record

**Figure 1:**
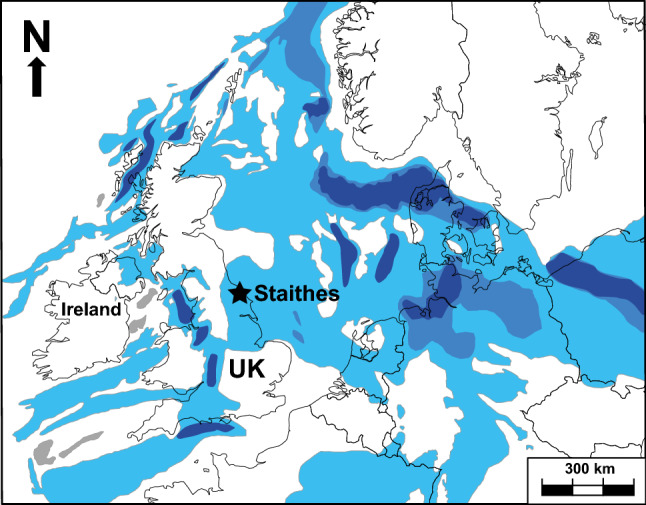
The location of the Staithes S-20 borehole displayed by the star, and the distribution of Permian–Triassic sedimentary basins of NW Europe marked by the blue tones. The darker tones represent thicker sedimentary sequences that accumulated along the main rift axes (adapted from Ref.^24^).

To address the lack of a single geographic and stratigraphic record, we have generated a high-resolution δ^34^S_evap_ curve from the Staithes S-20 borehole (NZ71NE/14; grid reference, NZ 476034E 518000N), Yorkshire, England (Fig. 1). The Staithes S-20 borehole was chosen due to its stratigraphic coverage (~ 668 m) of evaporite-bearing strata that are lithostratigraphically dated between the late Permian to Late Triassic. The Hardegsen unconformity has removed much of the Early Triassic in the Staithes S-20 borehole, although a palynological age constraint acquired from immediately above the unconformity is determined to be earliest Anisian in age (Warrington, *pers. comm.*, 2019; see Supplementary for more information).

A total of 364 individual evaporite samples (e.g., gypsum, anhydrite, and halite) were collected at regular intervals. For gypsum and anhydrite, a drill was used to produce a fine powder for isotopic analysis, whilst for halite the sulphate was obtained through barium sulphate precipitation (see Supplementary for Methodology).

**Figure 2:**
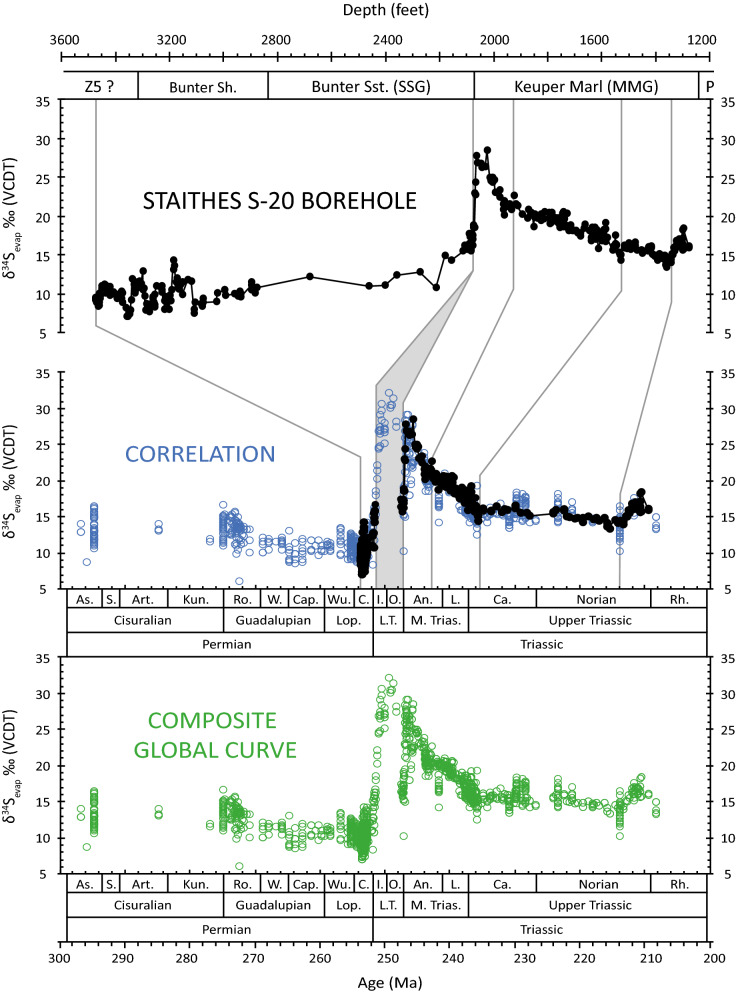
Sulphur isotope records derived from sedimentary evaporites from the Staithes S-20 borehole, northeast Yorkshire (top). The lithostratigraphy of the borehole is also displayed. The correlation between the global composite curve and the Staithes record (middle) is based primarily upon the trends and inflection points in the isotope records. The Staithes record was combined with the global record to produce a single composite curve (bottom; see Supplementary).

We compiled and recalibrated the global δ^34^S_evap_ curve for the Permian and Triassic, consisting of ~ 1000 δ^34^S_evap_ results (see Supplementary); our new, continuous record from a single site adds 38% more data to the global curve. All results were double-checked for their age assignment against a standardised geological timescale^25^. Based upon trends and inflection points in the global δ^34^S_evap_ record, we correlated the Staithes S-20 curve to generate a more robust global δ^34^S_evap_ record of the late Permian–Late Triassic; especially the Middle and Late Triassic (Fig. 2; see Supplementary).

## Late Permian: Early Triassic sulphur isotope instability

The composite late Permian—Early Triassic δ^34^S_evap_ record exhibits substantial variability (Fig. 2), interpreted as a product of environmental changes possibly induced by Siberian Traps volcanism^4^. The late Permian Zechstein evaporites have an average δ^34^S_evap_ of ~ 10.9‰, before lowering to ~ 8.2‰ at the PTB. Immediately following this, δ^34^S_evap_ values exhibit a sharp increase, reaching a maximum of ~ 32‰ at ~ 250 Ma in the Early Triassic (Fig. 2). Possibly facilitated by low sulphate concentrations^17,19^ due to deposition of the late Permian Zechstein evaporites^19^, this positive excursion reflects a major perturbation in the Early Triassic sulphur cycle. In addition, with the assistance of a palynological age constraint for the Hardegsen unconformity (see Supplementary), and stratigraphic correlation with the composite δ^34^S_evap_ curve, a new rapid negative δ^34^S_evap_ excursion (on the order of 15‰) is recorded at the Olenekian/Anisian boundary (OAB) (~ 247 Ma). Following this, the δ^34^S_evap_ record exhibits an abrupt recovery to pre-excursion values of 29‰ at ~ 246 Ma.

## Middle–Late Triassic sulphur isotope stability

The extreme environmental conditions that persisted during the late Permian and Early Triassic were more subdued in the Middle Triassic^15,17,19^. Accordingly, our δ^34^S_evap_ record exhibits a gradual and persistent decline from ~ 246 Ma in the early Anisian, before stabilising at ~ 236 Ma in the early Carnian (Fig. 2). Relative stability is maintained throughout the Carnian and the majority of the Norian.

Interestingly, we see no evidence for a substantial change in δ^34^S_evap_ during the Carnian Pluvial Event (CPE), potentially suggesting the environmental changes during the CPE had little impact on the global sulphur cycle. This is of interest, as the CPE is associated with major carbon cycle perturbations, the emplacement of the Wrangellian LIP (large igneous province) and a mass extinction event (followed by biotic radiation)^26,27^. It is thus intriguing that our δ^34^S_evap_ record maintains relative stability across this time interval. Higher resolution δ^34^S_evap_ records spanning the CPE, accompanied by further biogeochemical modelling, are required to confirm the apparent disconnect between the carbon and sulphur cycles during the CPE.

Our new δ^34^S_evap_ record also highlights the presence of a small positive δ^34^S_evap_ excursion (~ 4‰) prior to the Norian/Rhaetian boundary (Fig. 2), which potentially coincides with the emplacement of the Angayucham Complex (see below). Additional data are required to confirm the precise age and magnitude of this δ^34^S_evap_ excursion.

## Sulphur isotope box model and pyrite burial

**Figure 3:**
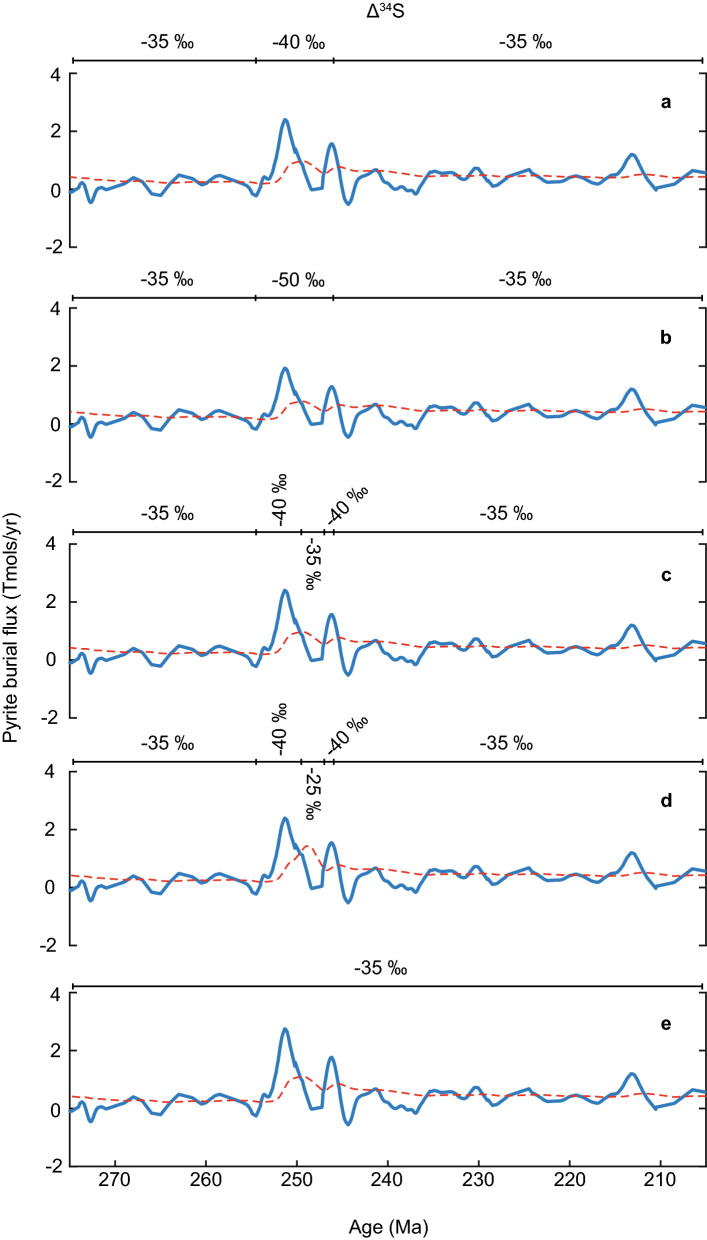
Sensitivity of the modelled pyrite burial flux to changes in the fractionation factor for the chemical reduction of sulphate to sulphide (Δ^34^S), and subsequent pyrite formation. The bar above each model output displays the value set for Δ^34^S at different intervals of time. The only parameter changed between each model run is Δ^34^S, enabling us to test the influence of this specific paramer on the inferred pyrite burial flux.

To explore the mechanisms responsible for the observed trends in the δ^34^S_evap_ curve, we incorporated our δ^34^S_evap_ data (compiled global dataset and the Staithes S-20 borehole data) into a sulphur isotope box model^28^ (see Supplementary). The model outputs predict three pyrite burial events (PBEs) during the time interval of this study, at ~ 251 Ma, ~ 246 Ma, and ~ 213 Ma (Fig. 3).

It should be noted however, that the fractionation factor (δ^34^S) associated with microbial reduction of sulphate to sulphide (and subsequent pyrite formation/burial) has been shown to vary according to a range of biological and environmental factors^29–31^. Recent biogeochemical modelling approaches suggest that variability in the δ^34^S of seawater sulphate during the Cenozoic can be accounted for by a shift in δ^34^S, reflecting a change in the locus of pyrite burial to deeper more oxygen-sparse water masses, rather than a simple change in pyrite burial rates^32^. Unfortunately, previous work^19^ did not consider a possible change in δ^34^S when interpreting variability in the δ^34^S of seawater sulphate observed for the Early Triassic.

**Figure 4:**
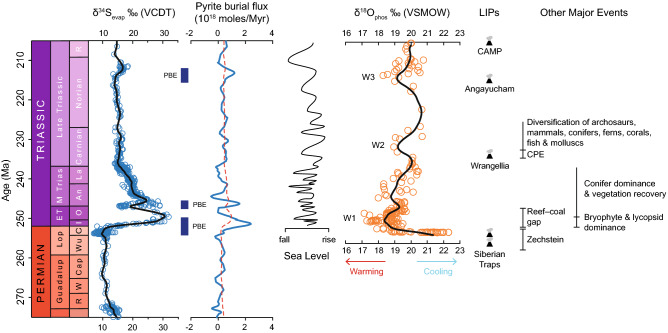
Sulphur and oxygen^8,18^ isotope records, modelled pyrite burial flux (see Fig. 3a) and environmental and biological changes for the latest Permian and Triassic. The sulphur and oxygen isotope data were fitted with a smoothing spline to produce the curves. The pyrite burial flux was calculated with the use of a sulphur isotope box model^28^ (see Supplementary). The predicted pyrite burial events are indicated with the abbreviation ‘PBE’. The blue line represents the calculated values, and the red dashed line illustrates the results assuming steady state. Environmental and biological events^43,44^ of significance are included to display how our isotope records and model outputs relate to the broader environmental context of the latest Permian and Triassic.

We completed a range of sensitivity tests to determine how shifts in the δ^34^S affected predicted pyrite burial rates (see Supplementary for details). We explored a range of values for δ^34^S between −35 and −50‰ for the Early Triassic (Fig. 3). Our results suggest that changing δ^34^S to more negative values supress the magnitude of the pyrite burial flux inferred for the PTB and earliest Triassic, but does not eliminate it entirely from the model outputs (Fig. 3). Thus, an increase in the magnitude of sulphur isotopic fractionation associated with pyrite formation is certainly possible, which is in line with evidence for an expansion of ocean anoxia during the PTB and Early Triassic time interval^13,33–35^. This may have contributed to the positive isotope excursion reported for the Early Triassic. However, our model outputs also predict that a change in δ^34^S within the range tested here would have been insufficient by itself to account for the positive shift in δ^34^S_evap_ during the Early Triassic. Thus, the Early Triassic δ^34^S_evap_ excursion must require an accompanying and substantial increase in the pyrite burial flux; a prediction in line with previous work^19,36^.It has been suggested that elevated CO_2_ and CH_4_ emissions associated with the Siberian Traps^5,7^ increased Earth’s surface temperature^8,18^. Along with the possible dieback of terrestrial vegetation^3^ and environmental acidity^37^, this likely increased continental weathering in the latest Permian and Early Triassic^9,37–39^. Weathering liberates bio-essential nutrients and may have heightened the supply of nitrogen and phosphorus to the surface oceans^33^, stimulating primary productivity^34,40^, and hence the flux of organic matter to the seafloor^34^. Oceanic oxygen solubility would have been low in a warm ocean, and combined with increased organic marine snow, this would have fuelled the expansion of anoxia/euxinia in the late Permian and Early Triassic^13,14,33^. Microbial sulphate reduction, encouraged by heightened nutrient fluxes and low oxygen concentrations would have driven the conversion of sulphate to sulphide and promote pyrite formation^41^ (and a “pyrite burial event”, PBE) in the presence of reduced iron. With the expansion of anoxia, pyrite formation may have occurred more readily within the water column^34,35,41^, heightening the magnitude of isotopic fractionation^31,42^. As suggested by our model results, this process would have sequestered isotopically light sulphur (^32^S) from the ocean reservoir, which would have contributed to the major positive δ^34^S_evap_ excursion in the Early Triassic (Fig. 4).

Our modelling outputs predict the subsequent negative δ^34^S_evap_ excursion at the OAB was preceded by a reduction in pyrite burial to a minimum of ~ –0.02 Tmol/year at 248 Ma (Fig. 4) (assuming a δ^34^S value of –40‰). As before, it was necessary to test for the sensitivity of inferred pyrite burial rates to changes in δ^34^S, and we thus completed several sensitivity tests with a range of values between –25 and –40‰ for the time interval 249 to 247 Ma (Fig. 3). For the above range of δ^34^S values, estimates for the pyrite burial flux minima at ~ 248 Ma varies between −0.03 and −0.02 Tmol/year, respectively. Thus, our modelling procedure suggests that the fractionation factor for sulphate reduction and pyrite formation had little control over the reduction in the pyrite burial flux across the OAB. The isotopic composition of pyrite (δ^34^S_pyr_) has been demonstrated to correlate with sea level fluctuations^30^, and is of interest considering the OAB coincides with a general fall in eustatic sea level^45^ (Fig. 4). It is intriguing that our modelling output suggests that changes in δ^34^S provide a relatively minor contribution to the decline in δ^34^S_evap_ values we report for the OAB. Therefore, this time interval may reflect the expansion of anoxia and shallowing of the chemocline^46^ inferred for much of the Early Triassic.

The available geochemical and sedimentological data fail to highlight any single mechanism for driving the observed negative δ^34^S_evap_ excursion, and therefore we propose several mechanisms.

Oxygen isotope data suggest a reduction in SSTs during the latest Spathian and early Anisian (Fig. 4)^8^. Cooling of marine waters would have likely been associated with invigoration of ocean circulation and lessened water column stratification^14^. Under such conditions, and in broad agreement with cerium-anomaly data for the latest Spathian^47^, the volume of anoxic water masses would have reduced, causing a decrease in pyrite burial (Fig. 4).

Coincident with the temperature decrease is a general fall in eustatic sea level^45^ that would have exposed either/or previously deposited (1) pyrite-rich shales from Early Triassic continental shelves) to weathering, (2) or extensive late Permian evaporite deposits (Zechstein). The sulphate released from pyrite oxidation and/or weathering of Permian Zechstein evaporites would be isotopically depleted (in comparison to Early Triassic δ^34^S values of + 32‰), thus contributing to the negative δ^34^S_evap_ excursion at the OAB. This is in line with our model outputs, which suggest a reduction in pyrite burial to ~ −0.02 Tmol/year (e.g., negative pyrite burial is equivalent to pyrite weathering because the model otherwise specifies constant pyrite weathering). Using either atmospheric oxygen and/or ferric iron as oxidants, the weathering of pyrite would yield sulphuric acid^48^, hence exacerbating weathering rates and contributing to the high ^87^Sr/^86^Sr values at the OAB^49^.

The recovery of δ^34^S_evap_ values to earliest Triassic levels of 29‰ immediately after the OAB is concomitant with an increase in the pyrite burial flux to ~ 1.54 Tmol/year at ~ 246 Ma (Fig. 4) (assuming a δ^34^S value of –40‰). We propose this reflects a recovery from the pyrite oxidation/evaporite weathering event responsible for causing negative δ^34^S_evap_ excursion at the OAB. In line with decreasing ^87^Sr/^86^Sr values in the early Anisian^39,49^, a relative decline in terrestrial weathering of sedimentary sulphides and evaporites would have reduced the flux of isotopically light sulphur into the ocean reservoir. In turn, this would have ensured rates of pyrite burial outpaced those of pyrite weathering, sequestering isotopically light sulphur from the seawater sulphate reservoir, facilitating a return to previous long-term δ^34^S_evap_ values (Fig. 4).

Although the predicted pyrite burial rates after the OAB return to positive values, they are lower than the Early Triassic peak (Fig. 4). This is to be expected, since predicted rates of pyrite burial began to decline prior to the weathering event at the OAB. This may indicate a gradual increase in sulphate concentrations and water column ventilation, in line with uranium isotope data that suggest a return to more oxygenated conditions in the early Anisian^50^. Although organic-rich claystones in the pelagic Panthalassic Ocean suggest deposition under anoxic conditions^34,51^, considering the uranium isotope record^50^, it is likely that anoxia was restricted to oxygen minimum zones and not the entire ocean as indicated for the earliest Triassic. In addition, our model outputs are based on long-term records and changes in the global δ^34^S_evap_ curve. Although it is likely that short-term events may coincide with minor changes in δ^34^S, our long-term δ^34^S_evap_ curve and box model outputs are insensitive to them.

Our Middle–Late Triassic δ^34^S_evap_ record from the Staithes S-20 core shows minimal variability around a consistent value of ~ 15‰ (Fig. 2); excluding δ^34^S_evap_ data that are grouped together from literature sources. In accordance with this, our pyrite burial model output also exhibits relative stability, with minor fluctuations around steady state (Fig. 4). The stabilisation observed in δ^34^S_evap_, and inferred for pyrite burial, is likely related to growth in the seawater sulphate reservoir^17,19^. Hence, more significant environmental perturbations would be required to disturb the global δ^34^S_evap_ record. The global, and long-term impact of the Siberian Traps would have ended, enabling the Earth’s climate system to re-establish more equable conditions^8^. Coincident with this, strontium isotope data show a general decline in the continental weathering flux^38,39^, thus reducing nutrient fluxes into the ocean, and stabilising the sulphur cycle^52^.

Global δ^34^S_evap_ data for the Late Triassic are sparse; therefore the δ^34^S_evap_ curve and model output rely heavily on the Staithes S-20 record. Towards the Norian/Rhaetian boundary there is a positive δ^34^S_evap_ excursion, which indicates an increase in pyrite burial from ~ 0.17 Tmol/year at ~ 217 Ma to ~ 1.2 Tmol/year at ~ 213 Ma (Fig. 4) (assuming a δ^34^S value of −35‰). Again, sensitivity tests were performed with a range of δ^34^S values between −25 and −50‰, yielding estimates for pyrite burial between 1.67 and 1.15 Tmol/year, respectively (see Supplementary). As before, shifting δ^34^S to more negative values reduced the magnitude of the predicted increase in pyrite burial; nonetheless, we still consider it a noteworthy PBE.

The precise mechanism behind this δ^34^S_evap_ excursion is currently unclear. A likely candidate is the emplacement of the Angayucham complex (Alaska, USA) at 214 ± 7 Ma^53^, which coincides with an oceanic warming event^18^, high CO_2_ concentrations^54^, and increasing humidity in Eastern Europe^55^ and the Alps^56^. Such environmental responses would have invigorated the hydrological cycle, thus increasing weathering and nutrient fluxes^57^, driving oceanic productivity in surface waters and oxygen consumption at depth in the water column. These environmental changes would have stimulated pyrite burial, and hence a positive δ^34^S_evap_ excursion. Tighter age constraint of the Angayucham Complex and additional δ^34^S_evap_ records over this time interval are necessary to ascertain their linkage. Why a δ^34^S_evap_ excursion is not present during a similar environmental event (CPE) is unclear and requires further investigation.

A direct comparison between LIP-induced environmental change in the geologic record and anthropogenic climate forcing is complex and ambitious. However, the fact that modern CO_2_ emissions are potentially 14 times greater than peak emission rates during the EPME^5,11^ is a matter of grave concern. The environmental changes recorded in our δ^34^S_evap_ record and the EPME lasted on the order of 10 million years before the sulphur and carbon biogeochemical cycles became stabilised. Current anthropogenic emissions have already shown a measurable impact on marine ecosystems globally^58^, a reduction in the pH of surface waters^59^, a decline in oxygen concentration^60^, and an increase in ocean stratification^61^. Understanding the long-term record of global Earth system perturbations caused by an elevation in greenhouse gases will improve our understanding of marine anoxia, weathering and pyrite burial events in the geologic record.

## Supplementary Information

Supplementary Information 1.

Supplementary Information 2.

## Data Availability

All data generated or analysed during this study are included in this published article (and its supplementary information files).
