# Supplementary material for: An 80-million-year sulphur isotope record of pyrite burial over the Permian–Triassic
Source: Sci Rep. 2022 Oct 17;12:17370. doi: 10.1038/s41598-022-21542-4 (PMC9576676; doi:10.1038/s41598-022-21542-4)
Supplement: Supplementary file 2 — Supplementary Information 2. [file 41598_2022_21542_MOESM2_ESM.pdf]

Supplementary Information

Contents

Regional geologic setting S2
Methods S4
Sampling S4
Sulphur isotope analysis of sulphate S4
Age assignment and data compilation S5
Preservation of geochemical signals S7
Model of the sulphur cycle S9
Supplementary figures S15
Supplementary tables S22
References S23

Regional geologic setting

Tectonic reconfiguration during the Palaeozoic and Mesozoic heralded the formation of the Pangaean supercontinent during the Variscan orogeny in the Late Carboniferous (Hounslow et al., 2012; Peacock, 2004). Rifting episodes began soon after in the Late Carboniferous with the propagation of the Norwegian-Greenland rift (Peacock, 2004), and continued during the Permian and Triassic, facilitating the development of linked fault-bound rift basins (Howard et al., 2008; McKie and Williams, 2009; Newell, 2018). These basins were separated by intrabasinal highs and massifs, and further subdivided by basement highs into an arrangement of sub-basins distributed throughout NW Europe (McKie, 2017; McKie and Williams, 2009). The formation of rift basins provided accommodation space for sediment, and seasonal monsoons sourced sediment for basin infills (McKie, 2017). Further rift propagation enhanced fault connectivity, providing marine waters episodic access to the intracratonic basins, facilitating evaporite deposition (McKie, 2017).

During the Late Permian and Triassic, Britain was located at a palaeolatitiude of ~20°N in eastern Pangaea (Newell, 2018). Deposition occurred under a dominantly arid climate regime throughout the Late Permian (McKie and Williams, 2009) due to the extent of continental aggregation and lack of open water bodies (McKie, 2017; Parrish, 1993). Prior to the Zechstein transgression, the Permian strata of Britain are characteristic of a desert depositional environment (Smith, 1989). In the Late Permian, tectonic adjustments in West Orkney and the Viking Graben opened a pathway between two rift structures through which the Boreal ocean gained periodic access to the subsiding Southern Permian Basin (SPB) (McKie, 2017; Smith, 1989). The Zechstein deposits that formed as a result are characterised by seven carbonate-evaporite sequences (Z1 through to Z7) (Tucker, 1991). The Early to Middle Triassic Sherwood Sandstone Group (SSG) is largely representative of a fluvial depositional regime, predominantly composed of brown, red, green/grey, and yellow arenaceous sandstones (Ambrose et al., 2014). It can be subdivided into different lithofacies, including fluvial channel facies, dryland terminal splay complexes, and mixed fluvial-aeolian facies (McKie, 2017; McKie and Williams, 2009). Braided river systems were fed by monsoonal rainfall on the Variscan mountains (Ambrose et al., 2014; Brookfield, 2008; Geluk et al., 2018), and were interrupted by periods of relative aridity that suppressed rates of fluvial sediment supply and facilitated aeolian reworking (McKie, 2017).

There is a cluster of late Early Triassic unconformities and disconformities identified across NW Europe that have been traced from Poland to Ireland and from onshore Germany/Netherlands into the southern and central North Sea (Bachmann et al., 2010, Bourquin et al., 2011). The largest of these occurs at the base of the Solling Formation in Germany and is variously described as the Base Solling or Hardegsen unconformity. The erosional truncation of stratigraphy (and merging of unconformities) is most pronounced on basin margins, such as the location of the Staithes S-20 well where a single truncation surface is preserved. In this onshore UK region Medici et al. (2019) estimate removal of at least 150 m of Early Triassic stratigraphy. The truncation appears to be gentle and can be traced from several hundred kilometres offshore to the east via the progressive removal of the Hardegsen and Detfurth formations in offshore regions and downcutting into the Early Triassic when approaching the onshore UK region (McKie, 2017).

Through the Early and Middle Triassic, the southern Permian basin was a uniformly and gently subsiding thermal sag basin with persistent stratigraphic motifs expressed on seismic by parallel reflectivity and persistent well log motifs that preserve their character over distances of hundreds of kilometres. Although individual wells tend to have sporadic biostratigraphic recovery the persistence of the stratigraphic units mean they can be traced between calibration points with high confidence. Tracing well logs from east to west the progressive truncation of the Bunter stratigraphy below the unconformity, and onlap of the Rot above allows the position of the unconformity in the Staithes S-20 well to be identified.

The SSG is overlain by the Middle–Late Triassic Mercia Mudstone Group (MMG), with the boundary being placed at the transition from sandstones to mudstones (Howard et al., 2008; Newell et al., 2018). The MMG is composed of green/grey mudstone interbedded with siltstone, with thick halite deposits and nodular calcium-sulphates — gypsum/anhydrite (Howard et al., 2008). Deposition and preservation of organic material is minimal in the strata of the UK Permian and Early Triassic. Accordingly, the evaporite samples from the Staithes S-20 borehole contain very little organic matter, and thus have no impact on the generation of the sulphur isotope data in this study (see below). Deposition coincided with the southerly retreat of the SSG beginning in the Middle Triassic, as fluvial systems were replaced by a hypersaline playa lake and mudflat environment (Howard et al., 2008). A greater marine influence during the deposition of Middle to Late Triassic deposits in NW Europe is likely (Newell et al., 2018). Evaporite deposition ceased during the Rhaetian coinciding with a marine transgression (Peacock, 2004) and the deposition of the Penarth Group (Gallois, 2009; Warrington and Ivimey-Cook, 1992). Biostratigraphic age constraints enable the Penarth Group to be

confidently assigned to the Rhaetian (Lott and Warrington, 1988; Hounslow and Ruffell, 2006), marking the transition to marine deposition that becomes well-developed in the Hettangian (Wignall and Bond, 2008; Gallois, 2009).

Methods

Sampling

364 evaporite samples were collected at regular intervals from the Boulby Mine, Staithes S-20 borehole (Grid Ref: 476024, 517997, Yorkshire, England. This borehole was chosen due to its stratigraphic coverage (~668 meters) of evaporite-bearing strata that is lithostratigraphically constrained from the late Permian to Late Triassic.

Sulphur isotope analysis of sulphate

Sulphur isotope analysis of evaporitic sulphate was performed on gypsum, anhydrite, and halite. Due to the high concentration of sulphate in gypsum and anhydrite (20 wt % sulphur, the process for sulphate extraction simply involves the use of a dentist's drill to produce a fine powder. In contrast, sulphate is only a trace constituent in halite and must be concentrated. For each sample, ~1–5 g of crushed halite was submerged in 30 ml of a 10 % solution of sodium chloride – blanks of NaCl produced no visible $BaSO_4$. The halite was left to sit in the solution for 24–48 hours, and agitated every few hours during the working day. Upon the dissolution of halite, the solution underwent centrifugation for 5 minutes at 3000 rpm, before the supernatant was decanted into a 50 ml centrifuge tube for subsequent barium sulphate ($BaSO_4$) precipitation. Approximately ~20 ml of barium chloride ($BaCl_2$) was mixed with 30 ml of the saline solution to extract the sulphate through the precipitation of barium sulphate, according to the following equation:

$$\mathrm{BaCl_2 + SO_4^{2-} \rightarrow BaSO_4 + 2Cl^{2-}} \quad [S1]$$

The pH of the solution was reduced to ~1–2 with the addition of 3M HCl to prevent any carbonate precipitation. The solution was left for >24 hrs to precipitate $BaSO_4$, after which it was centrifuged at 3000 rpm for 5 minutes. The supernatant was subsequently discarded for waste disposal and the $BaSO_4$ was rinsed with ~50 ml of deionised water to neutralise the acidity. After three rinses, the $BaSO_4$ was dried in an oven at 80°C for at least 24–48 hrs, and subsequently ground into a fine powder using an agate mortar and pestle.

For each sample, 0.3–0.5 mg was placed into tin capsules, with tungstic oxide (WO_3) added as a combustion-agent. Stable sulphur isotope analysis was performed in the Stable

Isotope Biogeochemistry Laboratory (SIBL) at Durham University using a Costech Elemental Analyser (ECS 4010) coupled to a Thermo Scientific Delta V Plus isotope-ratio mass-spectrometer. Evaporite sulphur isotope ratios are expressed as standard delta (δ) notation in per mil (‰) relative to the Vienna Canyon Diablo Triolite (VCDT) according to the following equation:

$$\delta^{34}\mathrm{S}_{\mathrm{evap}} = \left[\frac{(({}^{34}\mathrm{S}/{}^{32}\mathrm{S})_{\mathrm{sample}} - ({}^{34}\mathrm{S}/{}^{32}\mathrm{S})_{\mathrm{VCDT}})}{({}^{34}\mathrm{S}/{}^{32}\mathrm{S})_{\mathrm{VCDT}}}\right] * 1000 \quad [\mathrm{S2}]$$

The $\delta^{34}S$ data were normalised through calibration against four international standards (IAEA-S-1, IAEA-S-2, IAEA-S-3, NBS 127), providing a linear range in $\delta^{34}S$ between -32.49 ‰ and +22.62 ‰. Analytical uncertainty of $\delta^{34}S$ is typically ±0.2 ‰ for replicate analyses of the international standards. Total sulphur is calculated as part of the isotopic analysis using an internal standard, sulphanilamide (S = 18.619 wt %).

Age assignment and data compilation

The $\delta^{34}S_{evap}$ data of sulphate from Late Permian and Triassic marine evaporites include 1001 measurements from 54 references. Three published data compilations (Bernasconi et al., 2017; Crockford et al., 2019; Present et al., 2020) were used, and the age model of Bernasconi et al. (2017) was employed for the latest Permian and Triassic. Absolute age estimates from radiometric dating were maintained by the original source. However, absolute age estimates were sparse for Permian and Triassic data sets, with many studies relying upon lithostratigraphic and/or biostratigraphic age assignments. With this in mind, the age estimates were either maintained from the above compilations and/or adjusted based upon the most up-to-date available stratigraphic information. When adjustments to age were necessary, the International Commission on Stratigraphy 2020/03 (Cohen et al., 2013; updated) was used. However, if the sequence in question had biostratigraphic age constraints, the stratigraphic timescale of Ogg et al. (2016) was used instead.

Some studies included in their compilations, sulphur isotope values from aqueous brines that had acquired sulphate through the dissolution of local evaporite-bearing strata; we avoided such data, as it was difficult to confidently assign accurate age estimates for when that occurred. We have also excluded data from non-marine evaporites. However, if the sulphur isotope ratios (and the coupled oxygen isotope data when available) correlated well with coeval seawater $\delta^{34}S$ from other sources, the data was included even if the sedimentological and petrographic evidence suggested a non-marine and/or mixed marine/terrestrial origin. It should

be stressed that despite this, a cautious approach was taken when including published data, so if there was any uncertainty as to the source of the sulphate, the sulphur isotope data was not included.

When compiling our dataset, we tried to include sulphur isotope data that capture as broad a geographic coverage as possible. This was of course limited by the published data available, but was necessary to minimise the influence of local depositional and geochemical factors that may offset $\delta^{34}S$ from the global seawater $\delta^{34}S$ composition. Our compilation includes data from basins in northwest, central, southern, and eastern Europe, as well as Turkey, the Middle East, and North America. Admittedly, this presents a slight bias towards European basins.

The sulphur isotope analysis of 364 evaporite samples from the Staithes S-20 borehole, Yorkshire, England, provides one of the most high-resolution sulphur isotope curves for the latest Permian and Triassic. A sharp unconformity marking the boundary between the SSG and the MMG (Fig. S1) ensures that our record fails to capture much of the Early Triassic. Despite this unconformity, our $\delta^{34}S_{evap}$ record provides excellent stratigraphic coverage of the latest Permian and Triassic; sufficient for stratigraphic correlation. Biostratigraphic data are almost entirely absent, with the exception of one palynological age constraint just above the unconformity, dated to be earliest Anisian (Warrington, 2019, pers. comm.) (Fig. S2). Thus, correlation between the Staithes S-20 borehole and evaporite-bearing sequences globally is largely based upon the sulphur isotope data. Based upon general trends and inflection points in our record, we were able to correlate our $\delta^{34}S_{evap}$ curve from Staithes with our global composite record. Using this successful correlation, we divided the Staithes $\delta^{34}S_{evap}$ record according to the subdivisions of the Triassic. Thus, for the data within each subdivision, we derived lower and upper age estimates based upon the ages given to the subdivision boundaries. For each $\delta^{34}S_{evap}$ value, a numerical age estimate was then estimated based upon the depth of the associated sample relative to the thickness of strata within the relevant Triassic subdivision and the duration of time represented by such strata.

The palynology only provides an age estimate for strata above the unconformity, not below it. Thus, a biostratigraphic age constraint for the length of time represented by the unconformity is absent. This provides us with two options; the unconformity represents either a major time gap, or a relatively minor time gap (Fig. S3). We therefore determined two age models for the Staithes S-20 $\delta^{34}S_{evap}$ curve accordingly. One significant difference between the

two age models is the absence of an Early Triassic negative excursion if it is assumed the unconformity represents a minor time gap (Fig. S3B). If applied, this would have a major impact on our understanding of the response of the sulphur cycle to the End Permian Mass Extinction, and the results of the geochemical modelling. It is clear when viewing Figure S3A that a better correlation is attainable with the age model that assumes the unconformity represents a major time gap. In particular, according to Figure S3B, there is a large offset between the Staithes S-20 curve and the global composite for in the Early Triassic, in terms of both the timing and magnitude of the positive $\delta^{34}S_{evap}$ excursion.

It is also important to consider the sedimentology of the unconformity when assessing the suitability of either age model. The unconformity marks the boundary between the SSG and the MMG. It forms an abrupt transition from the arenaceous sandstone of the SSG and the Esk Evaporite Formation of the MMG (Fig. S1). We consider this change in lithology sufficient to support the assessment that the unconformity represents a major time gap (see discussion above). Thus, the sedimentological data, in conjunction with the correlation of the Staithes S-20 $\delta^{34}S_{evap}$ curve, suggests the age model including a major time gap in the Early Triassic is most appropriate.

Preservation of geochemical signals

The precipitation of gypsum and halite is associated with a fractionation factor that ensures a slight enrichment in the precipitate relative to the residual brine, between the range of 1.6 ‰ and 2.0 ‰ according to experimental studies (Holser and Kaplan, 1966; Nielsen, 1978; Raab and Spiro, 1991; Thode and Monster, 1965; Van Driessche et al., 2016). Beyond the halite stability field, an apparent change in the fractionation factor ensures a depletion of up to 4 ‰ in the precipitate relative to the residual brine (Raab and Spiro, 1991). Despite this, we are confident that the fractionation associated with mineral precipitation (below the halite stability field) has not obscured the primary seawater signal of our sulphur isotope record, as the isotopic enrichment of gypsum/anhydrite and halite is sufficiently small to be considered negligible in evaporite basins. In addition, we avoided sampling potash deposits, and focused entirely on calcium-sulphates and halite. Considering this, it is highly unlikely that the fractionation driven by evaporite mineral precipitation can explain the variability exhibited by our $\delta^{34}S_{evap}$ curve.

The sulphur isotope geochemistry of evaporites is considered less susceptible to diagenetic alteration than the carbonate equivalent, carbonate associated sulphate (CAS) (Bernasconi et al., 2017; Johnson et al., 2021). Despite this, evaporites are not entirely immune

to diagenesis (Schreiber and Tabakh, 2000), with microbes being capable of inducing changes in the geochemistry and mineralogy of evaporite deposits (Davis and Kirkland, 1979). For example, biologically-driven conversion of primary marine gypsum to elemental sulphur (Feely and Kulp, 1957) and the subsequent precipitation of secondary gypsum can be associated with the fractionation of sulphur isotopes (Feely and Kulp, 1957; Schreiber and Tabakh, 2000).

Calcium sulphates in evaporite deposits undergo a cycle of diagenesis during burial and uplift. Initially, primary gypsum precipitates from a saturated salt-water brine undergoing evaporation. Upon moderate to deep burial, gypsum dehydrates to replacement or pore-filling anhydrite. Subsequent uplift to near-surface depths can facilitate the rehydration of anhydrite to form secondary gypsum (Hardie, 1967; Murray, 1964; Ortí et al., 2022). In environments conducive to the precipitation of primary of very early diagenetic anhydrite, the cycle is limited to the rehydration of anhydrite to secondary gypsum (Ortí et al., 2022). As discussed previously, the initial formation of primary gypsum is accompanied by a negligible degree of sulphur isotope fractionation (Raab and Spiro, 1991). Unfortunately, due to the difficulties involved with precipitating primary anhydrite under experimental conditions, the associated fractionation remains poorly understood (Hardie, 1967; Ortí et al., 2022). Interestingly, data from the Khuff Formation, Abu Dhabi, suggests the dehydration of primary gypsum to anhydrite and thermochemical sulphate reduction at depth is associated with negligible sulphur isotope fractionation (Worden et al., 1997). The impact of the dehydration of diagenetic anhydrite on the sulphur isotopic composition of secondary gypsum has received little attention, and thus the fractionation factor is not well established (Ortí et al., 2022). However, studies analysing gypsum and anhydrite of equivalent age suggest no apparent offset in the isotopic composition between either mineral phase (Carrillo et al., 2014; Utrilla et al., 1992). Thus, we consider it unlikely that the Calcium sulphate diagenetic cycle obscured the primary seawater signal of the Ca-sulphates sampled as part of this study.

It should also be noted that evaporite deposition occurs in isolated basins with restricted access to the open ocean (Warren, 2010). Due to this, it is possible that the geochemistry of highly saline brines in evaporite basins does not reflect the geochemistry of seawater in the open ocean (Bernasconi et al., 2017), especially during periods of low seawater sulphate concentrations, which could ensure greater isotopic heterogeneity. Local sedimentary and geochemical processes, including riverine inputs, Rayleigh fractionation in a closed basin (Raab and Spiro, 1991), and the aforementioned microbial sulphur isotope fractionation, could

theoretically yield an isotopic offset between sulphate in an evaporite basin and the sulphate reservoir of the global ocean. Despite this, we consider it unlikely that local depositional and diagenetic effects had a significant impact on the trends exhibited by our global composite curve, or our $\delta^{34}S_{evap}$ record from the Staithes S-20 borehole. The data presented in our global curve exhibit a relatively small degree of scatter, and thus, we are confident that the consistent pattern of the $\delta^{34}S_{evap}$ measurements suggests that our global curve provides a global record for the latest Permian–Triassic seawater sulphate. The small degree of scatter may reflect minor influences from local effects, or possible difficulties with age constraint/correlation, but the average trend presents a robust primary signal. The record from the Staithes S-20 borehole exhibits no major, sudden shifts in $\delta^{34}S$, which suggests: (1) no significant unconformities (i.e., time gaps) except for that discussed above and in the main paper; (2) no diagenetic alteration of the sulphur isotope signature of these evaporites; (3) evaporite mineralogy had no effect on the $\delta^{34}S_{evap}$ curve; and (4) the evaporative basin in Yorkshire was still connected to the marine sulphate reservoir during the latest Permian and Triassic.

Model of the sulphur cycle

To explore the mechanisms responsible for the observed variability in the Triassic $\delta^{34}S$ curve, we employed the box model of Kurtz et al. (2003). This is a reverse-driven model and is controlled by our composite $\delta^{34}S_{evap}$ record. We establish boundary conditions (see Table S1), and the model yields an estimate for the pyrite burial flux with time (10^{18} moles S/m.y.).

The mass of sulphur in the ocean reservoir is controlled by the balance between the sulphur input fluxes and output burial fluxes (Gill et al., 2011). Biogeochemical perturbations that yield imbalances between the flux rates of sulphur can induce changes in the mass of marine sulphate over time. In the model, this relationship is represented by the following equation:

$$\frac{dM_0^S}{dt} = F_W^S - \left(F_{gyp} + F_{py}\right) \quad [S3]$$

The mass of marine sulphur is represented by M_0^S. The input fluxes of sulphur into the ocean reservoir via continental weathering and volcanic degassing are represented by a single variable, F_W^S, and set to a constant value (see Table S1). Output fluxes include the burial of gypsum (F_{gyp}) and pyrite (F_{py}).

Similarly, the isotopic composition of oceanic/atmospheric sulphate is primarily controlled by the respective contributions and isotopic composition of the sulphur fluxes entering and exiting the ocean reservoir (Bernasconi et al., 2017; Paytan et al., 2011). Therefore, variation in the stable sulphur isotopic composition of rocks and sediments is generally considered to be a product of perturbations in the biogeochemical cycling of sulphur (Richardson et al., 2019), such as changes in the rates of weathering and pyrite burial (Gill et al., 2007). This relationship is expressed in our model through the following equation, derived from multiplying the terms for the reservoir (i.e., its mass) and sulphur fluxes in Equation S3 by their isotopic compositions:

$$\frac{d}{dt}(M_0^S \delta_0^S) = F_W^S \delta_W^S - F_{gyp}\delta_0^S - F_{py}(\delta_0^S + \Delta_S) \quad [S4]$$

The sulphur isotopic composition of the marine reservoir is represented by δ_0^S. It should be noted that the minor fractionation associated with gypsum precipitation (Raab and Spiro, 1991) is ignored here, and thus δ_0^S also describes the isotope geochemistry of F_{gyp}. The burial of biomass associated organic sulphur, although significant in modern and ancient anoxic marine environments (Bauer et al., 2022), is accompanied by very little sulphur isotope fractionation (Anderson and Pratt 1995; Werne et al., 2003). Bauer et al. (2022) provide few constraints, with the authors simply invoking it as needed. As previously stated, our samples from the Staithes S-20 borehole contain very little organic material, preventing us from providing any constraints ourselves. The burial of biomass associated organic sulphur is thus considered, in the context of the model, to be isotopically indistinguishable from the burial of evaporites. In addition, the isotopic composition of sulphurised organic matter is comparable to sedimentary pyrite burial (although slightly more ^{34}S enriched) (Anderson and Pratt, 1995), and is important within anoxic water masses of modern and ancient marine environments (Raven et al., 2019; Bauer et al., 2022). However, due to the scarcity of organic material within the evaporites sampled from the Staithes S-20 borehole, no additional constraints are available to separate the two. Therefore, we have not included the sulphurisation of organic matter in our modelling procedure, as it would have limited to no impact on the output results. Although we encourage further research into this. Δ_S is the isotopic offset between seawater sulphate and sedimentary pyrite, induced during microbial sulphate reduction (MSR). The fractionation factor associated with MSR is far more significant than the fractionation during gypsum burial, and is thus set to values appropriate for the redox-state of the ocean (see Table S1). δ_W^S represents the sulphur isotopic composition of the riverine input flux. Derived from the

chemical weathering of material in the terrestrial reservoir, the isotopic signal is mixed between sulphur sourced from the weathering of evaporites and the oxidative weathering of sedimentary sulphides. Here we use a modern value of 4.8 ‰ (Burke et al., 2018) (Table S1), as we have no means for accurately estimating a value specific to the Triassic.

It was necessary for the model to estimate the change in the sulphur isotope geochemistry of the ocean reservoir for a given period of time without assuming steady-state conditions (Gill et al., 2011; Kurtz et al., 2003). To achieve this, Equation S3 was substituted into Equation S4 to derive Equation S5 as follows:

$$\frac{d\delta_0^S}{dt} = \frac{F_W^S(\delta_W^S - \delta_0^S) - F_{py}\Delta_S}{M_0^S} \quad [S5]$$

Where F_{gyp} has been omitted from Equation S5 for the reasons discussed above. Although Equation S5 can be solved for steady state, it does not necessarily assume such a condition. One assumption it does make, however, is homogeneity in the $\delta^{34}S$ of ocean sulphur at any one point in time (Kurtz et al., 2003).

Conditions of steady state ensure that there is no instantaneous change in the isotopic composition and mass of the sulphate reservoir in response to variations in the sulphur input flux, as the latter is balanced by complementary changes to the output fluxes of sulphur (Kurtz et al., 2003). However, due to the relatively long residence time of sulphur in the ocean (>10 Myrs) (Paytan et al., 2012), an assumption of steady-state is not appropriate when modelling the sulphur cycle over short periods of time, equal to, or less than 10 Myrs (Kurtz et al., 2003). This is significant, as some components of the sulphur isotope curve are characterised by rapid isotopic variability, with durations less than the residence time of marine sulphur. Thus, it was necessary for us to derive Equation S5 that does not necessarily assume steady state. This ensures that the model allows for adjustments in the mass and isotopic composition of the sulphur reservoir in response to changes in the input and output fluxes of sulphur (Kurtz et al., 2003). Equation S5 can be rearranged to Equation S6, which models the pyrite burial flux over a given period without assuming conditions of steady state:

$$F_{py} = \frac{F_W^S(\delta_W^S - \delta_0^S) - \frac{d\delta_0^S}{dt}M_0^S}{\Delta_S} \quad [S6]$$

Modelled rates of pyrite burial are subject to uncertainty because of the assumptions made during the modelling procedure, including variability in the degree of isotopic

fractionation and the continental weathering flux, both of which can influence the $\delta^{34}S$ of seawater sulphate. In addition, conditions of rapid isotopic change where steady state cannot be assumed, act to contribute to further uncertainty (Kurtz et al., 2003). To address this, it was necessary to complete a range of sensitivity tests to better constrain the true cause of the biogeochemical instability characterising our isotope record.

Changes in the degree of isotopic fractionation ($\Delta^{34}S$) during processes such as microbial sulphate reduction (MSR) can occur, and are constrained by a number of environmental and physiological factors (Canfield et al., 2010; Fike et al., 2015; Pasquier et al., 2017; Bryant et al., 2018; Rennie et al., 2018; Pasquier et al., 2021). In theory, if such changes did occur, this would influence the interpretation of our sulphur isotope record. Habicht et al. (2002) demonstrated that the fractionation factor for MSR is sensitive to ocean sulphate concentrations, and reported very low $\Delta^{34}S$ values. However, this is likely only the case under conditions of very low sulphate (<200 μM) characteristic of the Archean ocean, and is thus not likely to have had an impact on the isotopic variability of the Permian-Triassic $\delta^{34}S_{evap}$ record. We conducted a sensitivity test that enabled us to calculate the degree to which $\Delta^{34}S$ would have to vary to reasonably attribute the observed variability in our $\delta^{34}S_{evap}$ record to changes in $\Delta^{34}S$ alone. Figure S4 demonstrates that the Permian–Triassic $\delta^{34}S$ record would require an unrealistic degree of variability for $\Delta^{34}S$. Thus, we can say with relative confidence that $\Delta^{34}S$ did not exert a substantial influence on the $\delta^{34}S$ of Permian–Triassic seawater sulphate.

It is of course possible that changes in $\Delta^{34}S$, although alone not likely capable of inciting the variability observed in our $\delta^{34}S_{evap}$ record, may have been a contributing factor along with changes in the magnitude and isotopic composition of sulphur input fluxes, such as weathering and pyrite burial. We conducted additional sensitivity tests to assess modelled pyrite burial flux to the value set for $\Delta^{34}S$. For the Early Triassic positive $\delta^{34}S_{evap}$ excursion, increasing $\Delta^{34}S$ subdued the modelled pyrite burial flux predicted to have incited this excursion. Similarly, if the $\Delta^{34}S$ value is shifted to account for a reduction in the magnitude of isotopic fractionation during the time interval associated with the negative $\delta^{34}S_{evap}$ excursion, the magnitude of the predicted decrease in pyrite burial/increase in pyrite weathering is marginally subdued (Fig. 3 of main text). The implications of this are discussed further in the main text. We also applied this sensitivity test to the positive $\delta^{34}S_{evap}$ excursion near the Norian/Rhaetian boundary. For a range of $\Delta^{34}S$ values between -35 ‰ and -50 ‰: if the magnitude of fractionation is increased (i.e., $\Delta^{34}S$ becomes more negative), the predicted increase in pyrite burial necessary to account

for the positive excursion is lessened (Fig. S5). This suggests that although changes in $\Delta^{34}S$ may have contributed to driving the observed $\delta^{34}S_{evap}$ variability, accompanying changes in the rates of pyrite burial and weathering were likely required to account for the full extent of the isotopic variability of seawater sulphate during this time interval.

The sulphur isotopic composition of the riverine input flux ($\delta_W{}^S$) is constrained by the relative contributions from the terrestrial weathering of sedimentary pyrite and gypsum, which is largely unknown for any point in the geologic past (Kurtz et al., 2003). Due to the fractionation factor for the formation of pyrite, if all other parameters are held constant, a reduction in the sulphur weathering flux would yield an associated increase in the $\delta^{34}S$ of marine sulphate, assuming no coincident change in the pyrite burial flux (Kurtz et al., 2003). Therefore, under such conditions, an increase in the riverine input flux ($F_W{}^S$) would likely incite an isotopic enrichment of seawater sulphate. In this way, changes in the magnitude and isotopic composition of sulphate entering the ocean reservoir are capable of driving variability in the $\delta^{34}S$ of seawater sulphate (Fike et al., 2015).

To assess whether the magnitude of the riverine sulphur input flux could have exerted a dominant control over the variability present in the Permian–Triassic $\delta^{34}S_{evap}$ record, we rearranged Equation S4 to solve for $F_W{}^S$ and held the pyrite burial flux constant. Figure S6 shows that for the weathering flux to have a dominant control over the variability in the $\delta^{34}S_{evap}$ record during the Permian/Triassic boundary (PTB) and earliest Triassic, a preceding reduction in $F_W{}^S$ to -1.94 Tmol/yr at 252 Ma would be necessary. A reduction in weathering is in conflict with the available proxy data, which suggests a substantial increase in continental weathering rates during the latest Permian and Early Triassic (Sun et al., 2018; Korte et al., 2003). Considering this, and that negative weathering rates are mathematically equivalent to pyrite burial, the model outputs confirm that the weathering flux alone could not have incited the variability observed across the PTB and Early Triassic $\delta^{34}S_{evap}$ record. In contrast, the sensitivity test suggests weathering rates would need to rise to ~2.93 Tmol/yr at ~248 Ma to account for the negative $\delta^{34}S_{evap}$ excursion observed at the Olenekian/Anisian boundary (OAB). This is only marginally higher than the modern riverine sulphur flux derived from pyrite and sulphate weathering of ~2.8 Tmol/yr (Burke et al., 2018). Although we propose multiple possible mechanisms for the negative $\delta^{34}S_{evap}$ excursion at the OAB, this sensitivity test suggests an increase in sulphur weathering could have contributed to the variability in the sulphur isotopic composition of seawater sulphate across this time interval.

During the model runs, the riverine input flux (F_W^S) set to a chosen value. In this case we set the F_W^S to 1.5 Tmol/yr (Kump and Garrels, 1986; Kurtz et al., 2003; Gill et al., 2011; Owens et al., 2013). Research on the modern sulphur cycle presents an estimate for the modern value for F_W^S of ~2.8 Tmol/yr when anthropogenic contributions are not considered (Burke et al., 2018). An alternative estimate for F_W^S of 3.5 Tmol/yr was used by Rennie et al. (2018) when modelling the Cenozoic carbon and sulphur cycles. We thus considered it necessary to test for the sensitivity of our model to the value given to the riverine input flux. Raising the value to either 2.8 or 3.5 Tmol/yr changes the magnitude of the variability in pyrite burial necessary to account for the variability exhibited by the $\delta^{34}S_{evap}$ record (Fig. S7). Despite this, the trends in the record of inferred pyrite burial rates remain relatively unchanged, and thus although their magnitude is different, the number of pyrite burial events are not altered. This ensured that our overall interpretations are not impacted by a change in the value of F_W^S within the tested range.

To explore the sensitivity of the model outputs to ocean sulphate concentrations, the model was run assuming different values for the concentration of sulphate (Fig. S8). Three model runs were conducted for this test, the first assumed sulphate concentrations set to the values displayed in Table S1 and Fig. S9 (Fig. S8a), the second assumed a constant sulphate concentration of 12.5 mM (Fig. S8b) estimated for the Middle Triassic (Bernasconi et al., 2017), and third assumed a value of 28 mM (Fig. S8c), the modern concentration of seawater sulphate. As expected, the pyrite burial flux exhibits greater variability when sulphate concentrations are fixed at the Middle Triassic and modern values. A larger sulphate reservoir is less susceptible to perturbations, and thus larger changes in pyrite burial are required to incite the variability observed in the $\delta^{34}S_{evap}$ record.

Supplementary figures

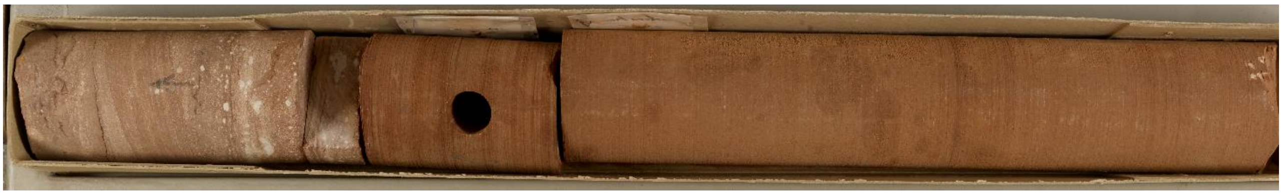

Figure S1: Cored material from Staithes S-20. The sharp unconformity can be seen separating the dark brown, laminated arenaceous sandstone of the Sherwood Sandstone Group, and the overlying gypsiferous silt-mudstone of the Mercia Mudstone Group (see arrow for way-up indicator).

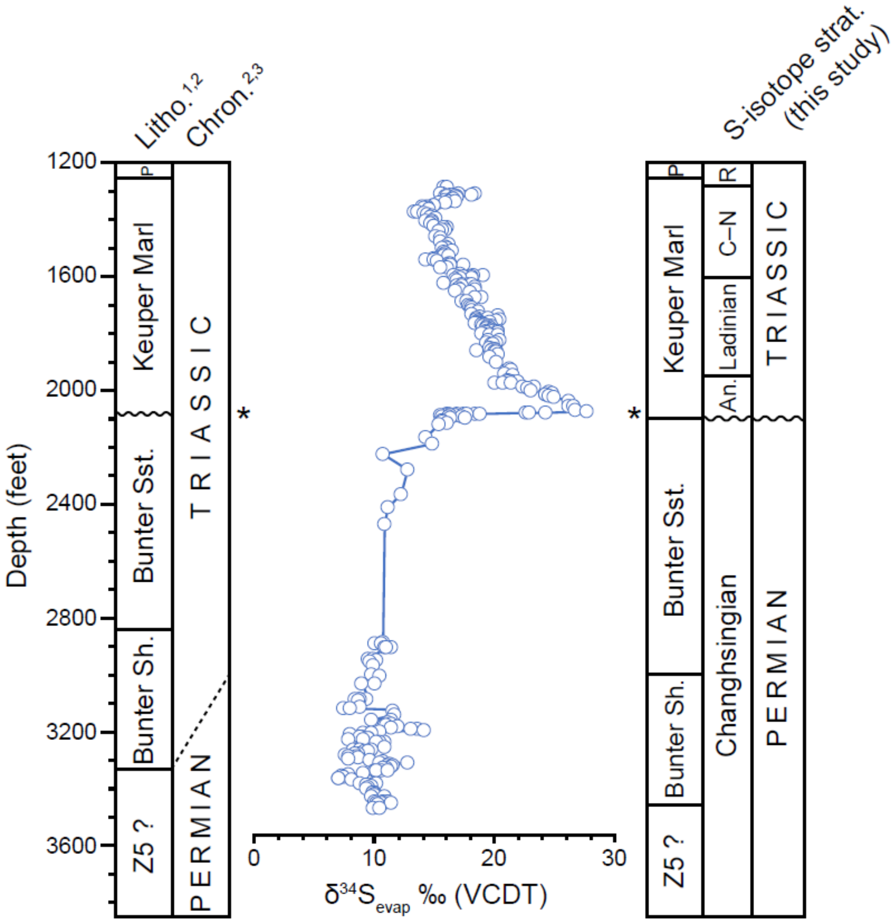

Figure S2: Staithes S-20 borehole $\delta^{34}S_{evap}$ record. References (1 = Woods, 1973; 2 = Jeans, 1995; 3 = Warrington, 2019, pers. comm.).

* = palynology sample assigned an earliest Anisian age (Warrington 2019, pers. comm.).

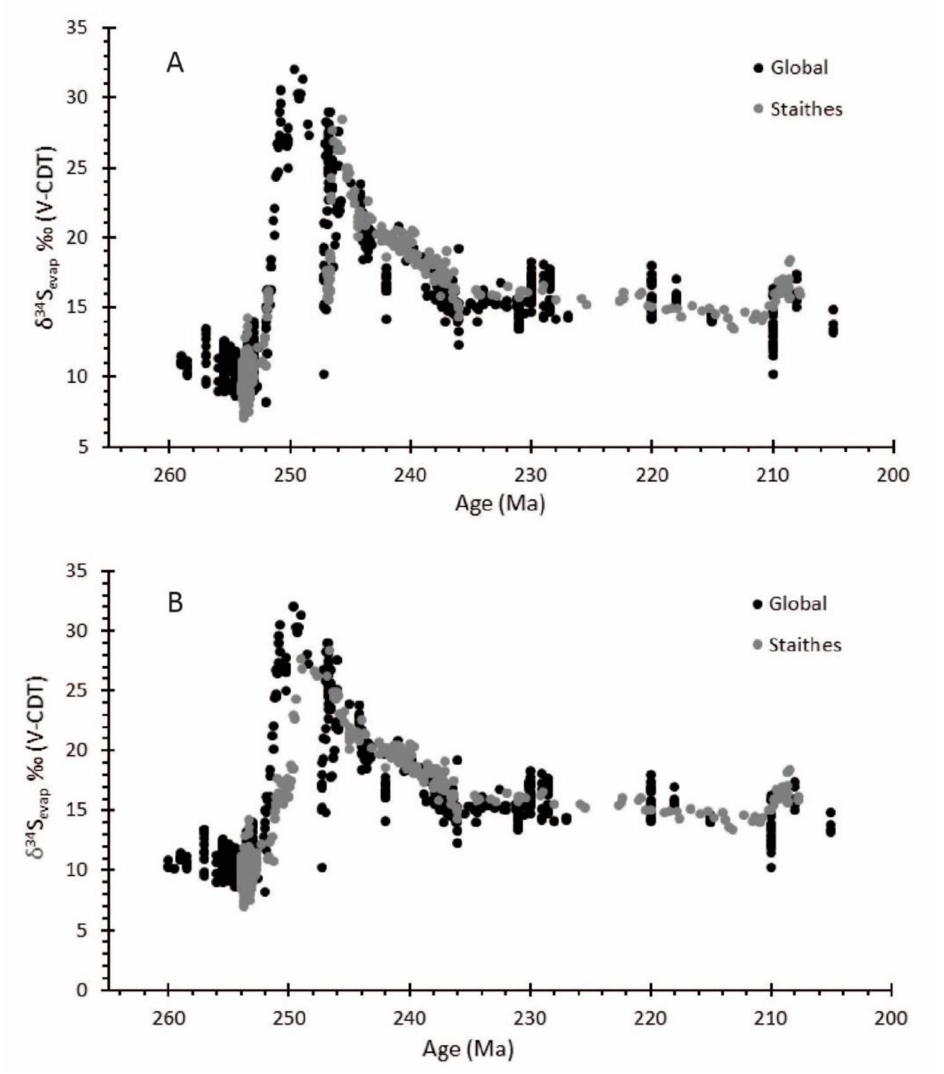

Figure S3: Comparison between the age model with a major time gap (A) and a minor time gap (B) based upon their correlation with the global composite $\delta^{34}S_{evap}$ curve.

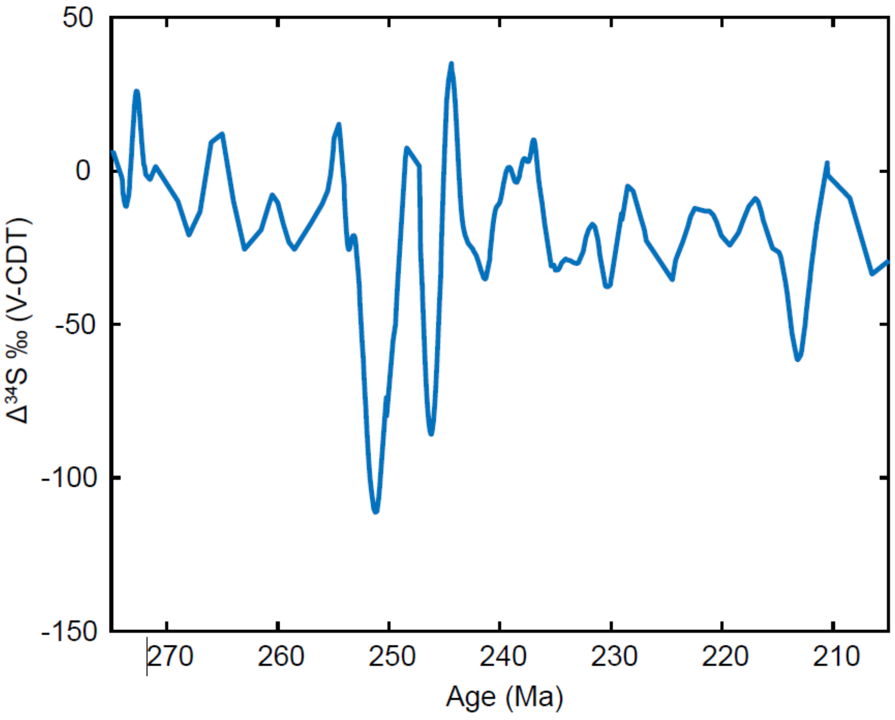

Figure S4: Modelled changes in $\Delta^{34}S$ required to incite the observed variability in the Permian–Triassic $\delta^{34}S$ record.

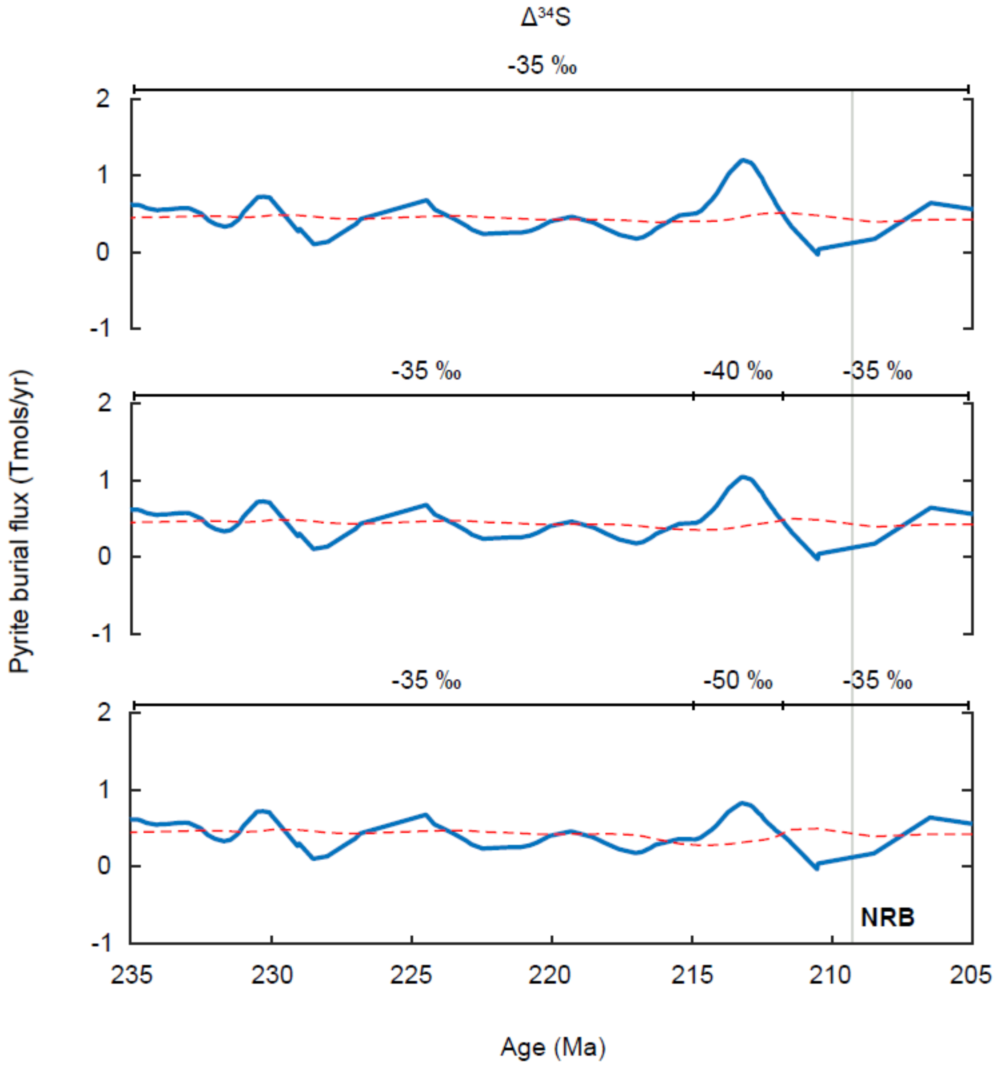

Figure S5: Sensitivity of the modelled pyrite burial flux to changes in the fractionation factor for the chemical reduction of sulphate to sulphide ($\Delta^{34}S$) during the Late Triassic. The bar above each model output displays the value set for $\Delta^{34}S$ at different intervals of time. The only parameter changed between each model run is $\Delta^{34}S$. The grey line marks the position of the Norian/Rhaetian boundary (NRB).

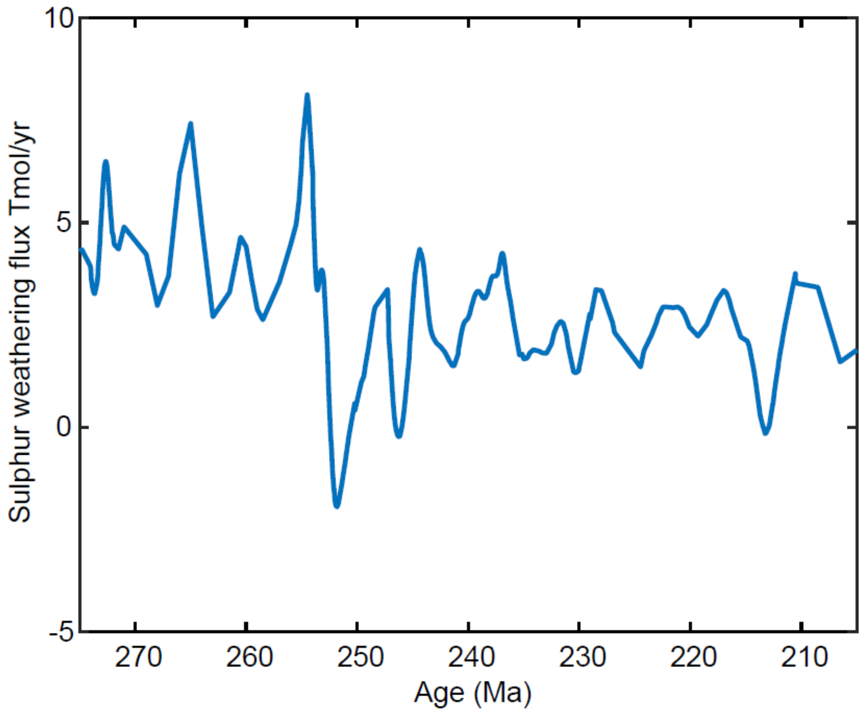

Figure S6: Modelled changes in the riverine sulphur weathering flux required to incite the observed variability in the Permian–Triassic $\delta^{34}S$ record.

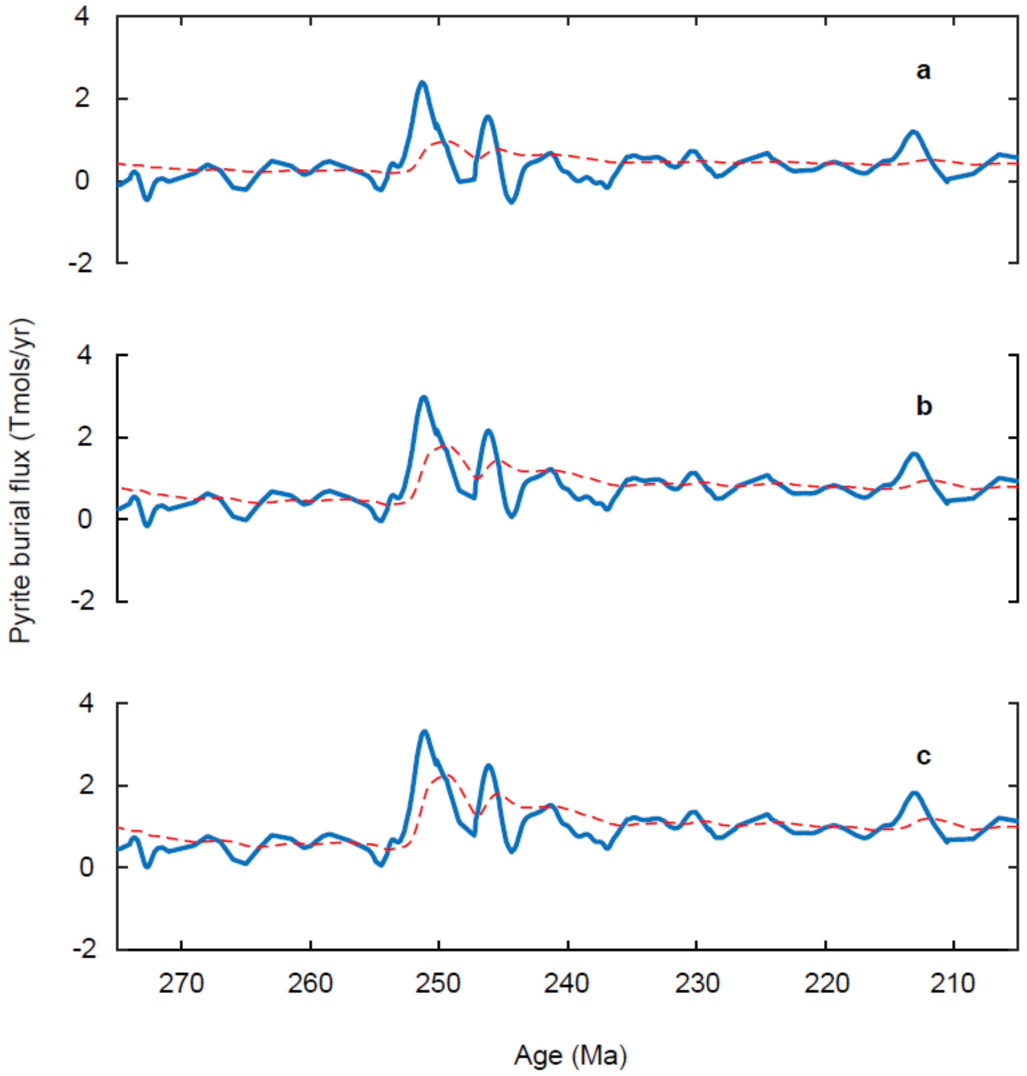

Figure S7: The sensitivity of the modelled pyrite burial flux to the value set for the weathering input flux (F_W^S). (a) displays the model output when F_W^S is set to 1.5 Tmol/yr (Kump and Garrels, 1986; Kurtz et al., 2003; Gill et al., 2011; Owens et al., 2013), and is the pyrite burial record used in the main text. (b) shows the inferred pyrite burial flux when F_W^S is set to the value estimated for the modern sulphur cycle of 2.8 Tmol/yr (Burke et al., 2018), and (c) displays the model output when F_W^S is set to 3.5 Tmol/yr (Rennie et al., 2018).

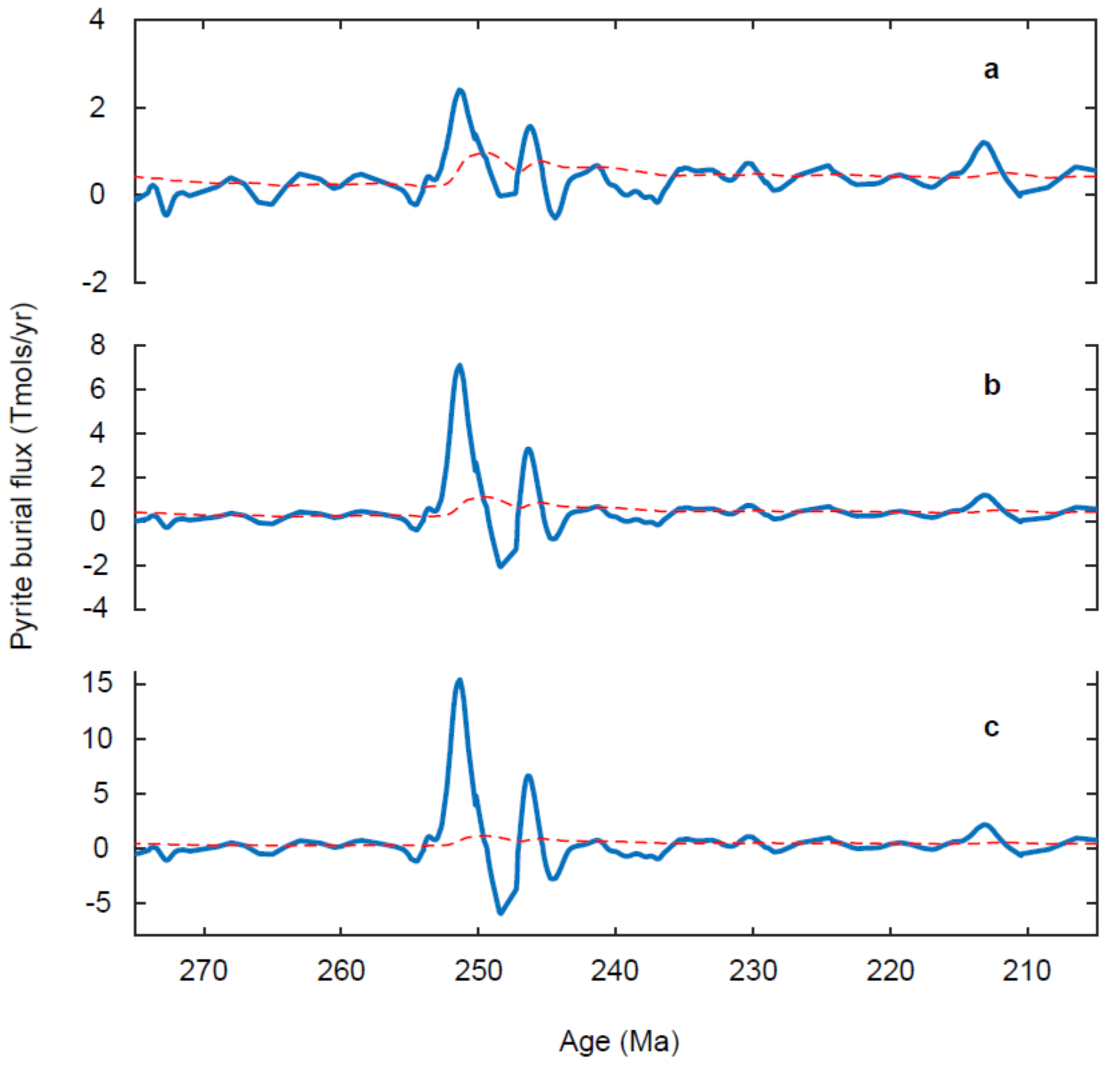

Figure S8: The sensitivity of the modelled pyrite burial flux to seawater sulphate concentration. The model was run assuming the sulphate concentrations (a) displayed in Table S1. A second model run (b) assumed the sulphate concentration was fixed at the value estimated for the Middle Triassic of 12.5 mM (Bernasconi et al., 2017), and a third (c) assumed the modern concentration of 28 mM. No other parameters were changed between model runs.

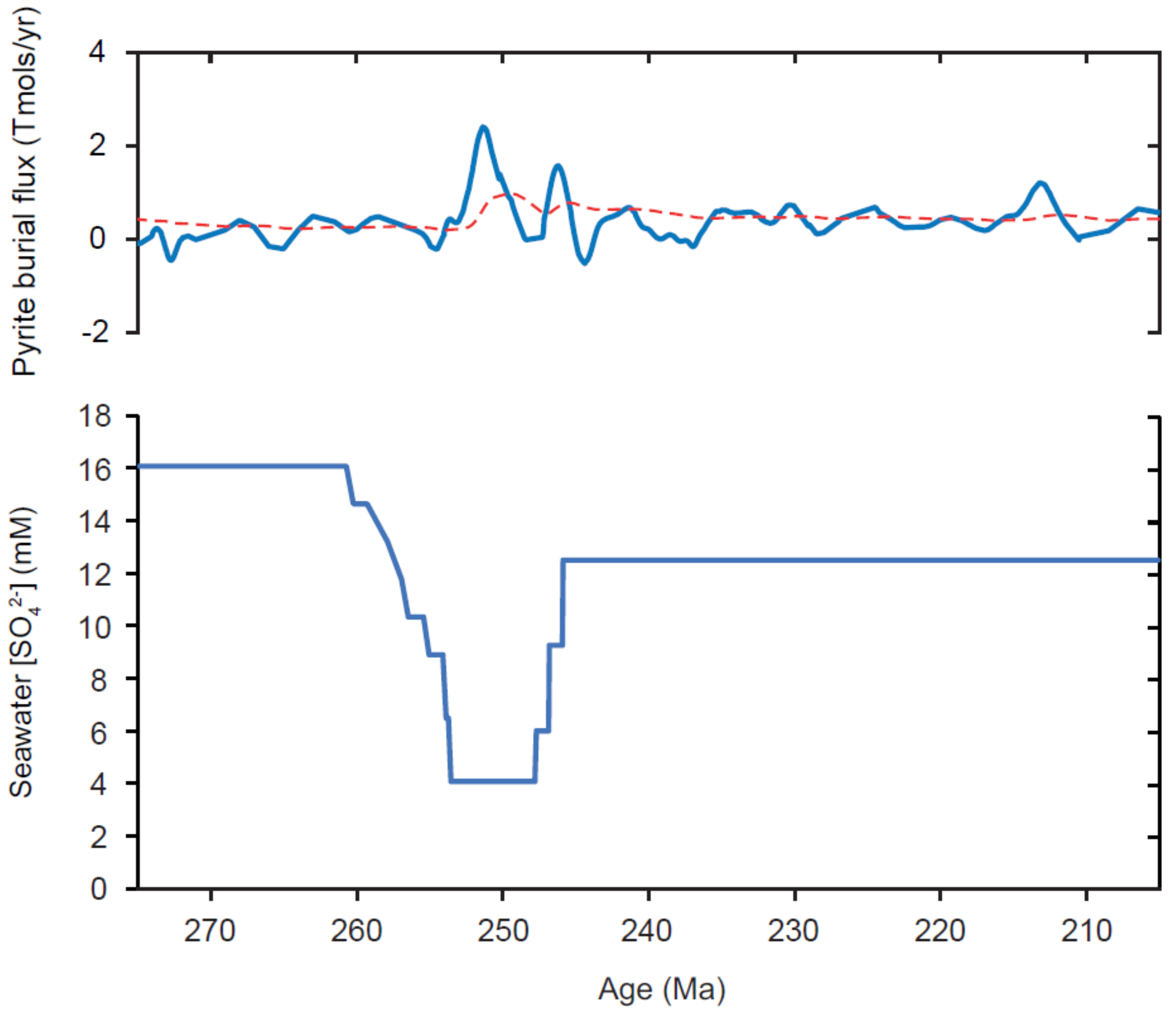

Figure S9: The pyrite burial flux predicted for the late Permian–Triassic plotted alongside seawater sulphate concentrations used during the modelling procedure. Unfortunately, high-resolution modelling only exists for the Early Triassic (Bernasconi et al., 2017; Stebbins et al., 2019). Horita et al. (2002) analysed fluid inclusions in halite to derive estimates for seawater sulphate concentrations for the late Permian. Few estimates exist for the Middle and Late Triassic, however Bernasconi et al. (2017) provide estimates for the early Middle Triassic. See Table S1 for the specific values used for seawater sulphate concentrations.

Supplementary tables

Parameter	Values applied	Source
M_O^S	Late Permian:<br>16 mM SO_4^{2-} (275–254 Ma)<br>8 mM SO_4^{2-} (254–252.5 Ma)<br><br>PTB through Early Triassic (252.5–246.3 Ma):<br>2–6 mM SO_4^{2-}<br><br>Early Middle Triassic (Early Anisian) (246.3–244 Ma):<br>6–9.3 mM SO_4^{2-}<br><br>Middle through Late Triassic (244–201 Ma):<br>10–15 mM SO_4^{2-}	Horita et al. (2002)<br><br><br><br>Bernasconi et al. (2017)<br><br><br><br><br><br>Bernasconi et al. (2017)
F_W^S	$1.5x10^{18}$mol m.yr.	Kurtz et al. (2003)
δ_W^S	4.8 ‰	Burke et al. (2018)
Δ_S	-35 ‰<br>-40 ‰ for anoxia	Gill et al. (2011)<br>Owens et al. (2013)
F_{gyp}	$0.5x10^{18}$ mol Myr	Gill et al. (2011)
F_{py}	$1.0x10^{18}$ mol Myr	Gill et al. (2011)

Table S1: Values for parameters used in the sulphur isotope box model.

References

Ambrose, K., Hough, E., Smith, N.J.P., Warrington, G., 2014. Lithostratigraphy of the Sherwood Sandstone Group of England, Wales and south-west Scotland. *British Geological Survey Research Report*, RR/14/01

Anderson, T.F., Pratt, L.M., 1995. Isotopic evidence for the origin of organic sulfur and elemental sulfur in marine sediments. In: Vairavamurphy, M.A., Schoonen, M.A.A., (eds) *Geochemical transformations of sedimentary sulfur, ACS symposium series*, vol., 612, pp., 378-396

Ault.W.U, Kulp.J.L, 1959. Isotopic geochemistry of sulfur, *Geochimica et Cosmochimica Acta*, vol 16(4), pp. 201-235

Bachmann, G.H., Geluk, M.C., Warrington, G., Becker-Roman, A., Beutler, G., Hagdorn, H., Hounslow, M.W., Nitsch, E., Röhling, H.-G., Simon, T., Szulc, A., 2010. Triassic. In: Doornenbal, J.C., Stevenson, A.G. (eds) *Petroleum Geological Atlas of the Southern Permian Basin Area*. EAGE Publications b.v. (Houten), pp., 149-173

Baure, K.W., Bottini, C., Katsev, S., Jellinek, M., Francois, R., Erba, E., Crowe, S.E., 2022. Ferruginous oceans during OAE1a and collapse of the marine sulfate pool. *Earth and Planetary Science Letters*, vol., 578, pp., 117324

Bernasconi, S.M., Meier, I., Wohlwend, S., Brack, P., Hochuli, P.A., Blasi, H., Wortmann, U.G., Ramseyer, K., 2017. An evaportite-based high-resolution sulfur isotope record of Late Permian and Triassic seawater sulfate, *Geochimica et Cosmochimica Acta*, vol. 204, pp.331-349

Bojar.A.V, Halas.S, Bojar.H.P, Trembaczowski.A, 2016. Late Permian to Triassic isotope composition of sulfates in the Eastern Alps: palaeogeographic implications, *Geological Magazine*, vol 155(4), pp.1-14

Bourquin, S., Bercovici, A., López-Gómez, J., Diez, J.B., Broutin, J., Ronchi, A., Durand, M., Arché, A., Linol, B., Amour, F., 2011. The Permian-Triassic transition and the onset of Mesozoic sedimentation at the northwestern peri-Tethyan domain scale: Palaeogeographic maps and geodynamic implications. *Palaeogeography, Palaeoclimatology, Palaeoecology*, vol., 299, pp., 265-280

Brookfield, M.E., 2008. Palaeoenvironments and palaeotectonics of the arid to hyperarid intracontinental latest Permian-Late Triassic Solway Basin (U.K.). *Sedimentary Geology*, vol., 210, pp., 27-47

Bryant, R.N., Jones, C., Raven, M.R., Gomes, M.L., Berelson, W.M., Bradley, A.S., Fike, D.A., 2018. Sulfur isotope analysis of microcrystalline iron sulfides using secondary ion mass spectrometry imaging: Extracting local paleo-environmental information from modern and ancient sediments. *Rapid Communications in Mass Spectrometry*, vol., 33, pp., 491-502

Burke, A., Present, T.M., Paris, G., Rae, E.C.M., Sandilands, B.H., Gaillardet, J., Peucker-Ehrenbrink, B., Fischer, W.W., McClelland, J.W., Spencer, R.G.B., Voss, B.M., Adkins, J.F., 2018. Sulfur isotopes in rivers: Insights into global weathering budgets, pyrite oxidation, and the modern sulfur cycle. *Earth and Planetary Science Letters*, vol., 496, pp., 168-177

Butcher.G.S, Kendall.A.C, Boyce.A.J, Millar.I.L, Andrews.J.E, Dennis.P.F. 2012. Age determination of the Lower Watrous red-beds of the Williston Basin, Saskatchewan, Canada, *Bulletin of Canadian Petroleum Geology*, vol 60(4), pp.227-238

Carrillo, E., Rosell, L., Ortí, F., 2014. Multiepisodic evaporite sedimentation as an indicator of palaeogeographic evolution in foreland basins (South-eastern Pyrenean basin, Early-Middle Eocene). *Sedimentology*, vol., 61, pp., 2086-2112

Canfield, D.E., Farquhar, J., Zerkle, A.L., 2010. High isotope fractionations during sulfate reduction in a low-sulfate euxinic ocean analog. *Geology*, vol., 38, pp., 415-418

Claypool.G.E, Holser.W.T, Kaplan.I.R, Sakai.H, Zak.I, 1980. The age curves of sulfur and oxygen isotopes in marine sulfate and their mutual interpretation, *Chemical Geology*, vol 28, pp.199-260

Clement.G.P, Holser.W.T, 1988. Geochemistry of Moroccan evaporites in the setting of the North Atlantic Rift, *Journal of African Earth Sciences*, vol 7(2), pp.375-383

Clemmensen.L, Holser.W.T, Winter.D, 1985. Stable isotope study through the Permian-Triassic boundary in East Greenland, *Bulletin of the Geological Society of Denmark*, vol 33, pp.253-260

Cohen, K.M., Finney, S.M., Gibbard, P.L., Fan, J.X., 2013. The ICS International Chronostratigraphic Chart. *Episodes 36*, 199-204.

Crockford, P.W., Kunzmann, M., Bekker, A., Hayles, J., Bao, H., Halverson, G.P., Peng, Y., Bui, T.H., Cox, G.M., Gibson, T.M., Wöndle, S., Rainbird, R., Lepland, A., Swanson-Hysell, N.L., Master, S., Sreenivas, B., Kuznetsov, A., Krupenik, V., Wing, B.A., 2019. Claypool continued: Extending the isotopic record of sedimentary sulfate, *Chemical Geology*, vol. 513, pp.200-225

Davis, J.B., Kirkland, D.W., 1979. Bioepigenetic sulphur deposits. *Economic Geology*, vol. 74, pp.462-468

Denison.R.E, Kirkland.D.W, Evans.R, 1998. Using strontium isotopes to determine the age and origin of gypsum and anhydrite beds, *The journal of Geology*, vol 106, pp.1-17

Dill.H.G, Bechtel.A, Berner.Z, Botz.R, Kus.J, Heunisch.C, Abu Hamad.A.M.B, 2012. The evaporite-coal transition: Chemical, mineralogical and organic composition of the Later Triassic Abu Ruweis Formation, NW Jordan – Reference type of the “Arabian Keuper”, *Chemical Geology*, vol 298, pp.20-40

Erkan.E, 1989. Die Sulfatlagerstätten der postvariszischen Transgressionsserie in den Ostalpen, *Nachrichten der Deutschen Geologischen Gesellschaft*, vol 41, pp.90-91

Feely, H.W., Kulp, J.L., 1957. Origin of Gulf Coast salt-dome sulphur deposits. *AAPG Bulletin*, vol. 41(8), pp.1802-1853

Fike, D.A., Bradley, A.S., Rose, C.V., 2015. Rethinking the ancient sulfur cycle. *Annual Review of Earth and Planetary Sciences*, vol., 43, pp., 593-622

Galamay.A.R, Meng.F, Bukowski.K, Ni.P, Shanina.S.N, Ignatovich.O.O, 2016. The sulphur and oxygen isotopic composition of anhydrite from the Upper Pechora Basin (Russia): new data in the context of the evolution of the sulphur isotopic record of Permian evaporites, *Geological Quarterly*, vol 60(4), pp.990-999

Gallois, R.W., 2009. The lithostratigraphy of the Penarth Group (Late Triassic) of the Severn Estuary area. *Geoscience in South-West England*, vol. 12, pp.71-84

García-Veigas.J, Cendón.D.I, Pueyo.J.J, Peryt.T.M, 2011. Zechstein saline brines in Poland, evidence of overturned anoxic ocean during the late Permian mass extinction event, *Chemical Geology*, vol 290, pp.189-201

García-Veigas.J, Cendón.D.I, Rosell.L, Ortí.F, Ruiz.J.T, Martín.J.M, Sanz.E, 2013. Salt deposition and brine evolution in the Granada Basin (Late Tortonian, SE Spain), *Palaeogeography, Palaeoclimatology, Palaeoecology*, vol 369, pp.452-465

Geluk, M., McKie, T., Kilhams, B., 2018. An introduction to the Triassic: current insights into the regional setting and energy resource potential of NW Europe. In: Kilhams, B., Kukla, P.A., Mazur, S., McKie, T., Mijnlieff, H.F., van Ojik, K. (eds) *Mesozoic Resource Potential in the Southern Permian Basin*. Geological Society, London, Special Publications, vol., 469, pp., 139-147

Gibert.L, Ortí.F, Rosell.L, 2007. Plio-Pleistocene lacustrine evaporites of the Baza Basin (Betic Chain, SE Spain), *Sedimentary Geology*, vol 200, pp.89-116

Gill, B.C., Lyons, T.W., Saltzman, M.R., 2007. Parallel, high-resolution carbon and sulfur isotope records of the evolving Paleozoic marine sulfur reservoir. *Palaeogeography, Palaeoclimatology, Palaeoecology*, vol. 256, pp.156-173

Gill, B.C., Lyons, T.W., Young, S.A., Kump, L.R., Knoll, A.H., Saltzman, M.R., 2011. Geochemical evidence for widespread euxinia in the Later Cambrian ocean. *Nature*, vol. 469, pp.80-83

Götzinger.M.A, Pak.E, 1983. Zur schwefelisotopen-verteilung in sulfid- und sulfatmineralen triadischer Gesteine der Kalkalpen, Österreich, *Mitteilungen der Gesellschaft der Geologie- und Bergbaustudenten Österreichs*, vol 29, pp.191-198

Gündoğan.I, Helvaci.E, Sözbilir.H, 2008. Gypsiferous carbonates at Honaz Daği (Denizli): First documentation of Triassic gypsum in western Turkey and its tectonic significance, *Journal of Asian Earth Sciences*, vol 32, pp.49-65

Habicht, K.S., Gade, M., Thamdrup, B., Berg, P., Canfield, D.E., 2002. Calibration of sulfate levels in the Archean ocean. *Science*, vol., 298, pp., 2372-2374

Hardie, L.A., 1967. The gypsum-anhydrite equilibrium at one atmosphere pressure. *The American Mineralogist*, vol., 52, pp., 171-200

Holser.W.T, Clement.G.P, Jansa.L.F, Wade.J.A, 1988. Evaporite deposits of the North Atlantic Rift. In: Manspeizer.W. (ed) *Triassic-Jurassic rifting: continental breakup and the origin of the Atlantic Ocean and passive margins: Developments in Geotectonics Parts A and B*. Elsevier, Amsterdam, pp.525-556

Holser, W.T., Kaplan, I.R., 1966. Isotope geochemistry of sedimentary sulfates, *Chemical Geology*, vol. 1, pp.93-135

Holser.W.T, Magaritz.M, 1985. The Late Permian carbon isotope anomaly in the Bellephoron Basin, Carnic and Dolomite Alps, *Jahrbuch der Geologischen Bundesanstalt*, vol 128(1), pp.75-82

Horita, J., Zimmermann, H., Holland, H.D., 2002. Chemical evolution of seawater during the Phanerozoic: Implications for the record of marine evaporites. *Geochimica et Cosmochimica Acta*, vol., 66(21), pp., 3733-3756

Hounslow, M.W., McKie, T., Ruffell, A.H., 2012. Permian to Late Triassic post-orogenic collapse and rifting, arid deserts, evaporating seas and mass extinctions. In: Woodcock, N.H., Strachan, R.A (eds) *The Geological History of Britain and Ireland*, 2nd revised edn, Wiley, Chichester, pp.301-321

Hounslow, M., Ruffell, A.H., 2006. Triassic: Seasonal rivers, dusty deserts and saline lakes. In: Rawson, P.F., Brenchley, P. (eds) *The Geology of England and Wales*, Geological Society of London, pp., 295-325

Hovorka.S.D, Knauth.L.P, Fisher.R.S, Gao.G, 1993. Marine to nonmarine facies transition in Permian evaporites of the Palo Duro Basin, Texas: Geochemical response, *Geological Society of America Bulletin*, vol 105, pp.1119-1134

Howard.A.S., Warrington.G., Ambrose.K., Rees.J.G., 2008. A formational framework for the Mercia Mudstone Group (Triassic) of England and Wales. *British Geological Survey Research Report*, RR/08/004

Hryniv.S.P, Peryt.T.M, 2003. Sulfate cavity filling in the Lower Werra Anhydrite (Zechstein, Permian), Zdrada Area, Northern Poland: Evidence for early diagenetic evaporite paleokarst formed under sedimentary cover, *Journal of Sedimentary Research*, vol 73(3), pp.451-461

Huerta.P, Armenteros.I, Recio.C, Blanco.J.A, 2010. Palaeogroundwater evolution in playa-lake environments: Sedimentary facies and stable isotope record (Palaeogene, Almazán basin, Spain), *Palaeogeography, Palaeoclimatology, Palaeoecology*, vol 286, pp.135-148

Jeans, C.V., 1995. Clay mineral stratigraphy in Paleozoic and Mesozoic red bed facies onshore and onshore UK. In: Dunay, R.E., Hailwood, E.A., (ed) *Non-biostratigraphical methods of dating and correlation*, Geological Society Special Publication No., 89, pp., 31-55

Johnson, D.L., Present, T.M., Li, M., Shen, Y., Adkins, J.F., 2021. Carbonate associated sulfate (CAS) $\delta^{34}S$ heterogeneity across the End-Permian Mass Extinction in South China. *Earth and Planetary Science Letters*, vol., 574, pp., 117172

Kovalevych.V, Peryt.T.M, Beer.W, Geluk.M, Halas.S, 2002. Geochemistry of Early Triassic seawater as indicated by study of the Röt halite in the Netherlands, Germany, and Poland, *Chemical Geology*, vol 182(2-4), pp.549-563

Kramm.U, Wedepohl.K.H, 1991. The isotopic composition of strontium and sulfur in seawater of Late Permian (Zechstein) age, *Chemical Geology*, vol 90, pp.253-262

Kump, L.R., Garrels, R.M., 1986. Modeling atmospheric O_2 in the global sedimentary redox cycle. *American Journal of Science*, vol., 286, pp., 337-360

Kurtz, A.C., Kump, L.R., Arthur, M.A., Zachos, J.C., Paytan, A., 2003. Early Cenozoic decoupling of the global carbon and sulfur cycles. *Paleoceanography*, vol. 18(4)

Lepetit.P, Aehnelt.M, Viereck.L, Strauss.H, Abratis.M, Fritsch.S, Malz.A, Kukowski,N, Totsche.K.U, 2019. Intraformational fluid flow in the Thuringian Syncline (Germany) – Evidence from stable isotope data in vein mineralization of Upper Permian and Mesozoic sediments, *Chemical Geology*, vol 523, pp.133-153

Leslie.A.B, Harwood.G.M, Kendall.A.C, 1997. Geochemical variations within a laminated evaporite deposit: evidence for brine composition during formation of the Permian Castile Formation, Texas and New Mexico, USA, *Sedimentary Geology*, vol 110, pp.223-235

Lott, G.K., Warrington, G., 1988. A review of the latest Triassic succession in the U.K. sector of the Southern North Sea Basin. *Proceedings of the Yorkshire Geological Society*, vol., 47(2), pp., 139-147

Marenco.P.J, Corsetti.F.A, Kaufman.A.J, Bottjer.D.J, 2008. Environmental and diagenetic variations in carbonate associated sulfate: An investigation of CAS in the Lower Triassic of the western USA, *Geochimica et Cosmochimica Acta*, vol 72, pp.1570-1582

McKie, T., 2017. Paleogeographic evolution of latest Permian and Triassic salt basins in Northwest Europe. In: Soto, J.I., Flinch, J.F., Tari, G. (eds) *Permo-Triassic salt provinces of Europe, North Africa and the Atlantic Margins, tectonics and hydrocarbon potential*, pp., 159-173

McKie, T., Williams, B., 2009. Triassic palaeogeography and fluvial dispersal across the northwest European Basins. *Geological Journal*, vol., 44, pp., 711-741

Medici, G., West, L.J., Mountjoy, N.P., 2019. Sedimentary flow heterogeneities in the Triassic U.K. Sherwood Sandstone Group: Insights for hydrocarbon exploration. *Geological Journal*, vol., 54(3), pp., 1361-1378

Murray, R.C., 1964. Origin and diagenesis of gypsum and anhydrite. *Journal of Sedimentary Petrology*, vol., 34, pp., 512-523

Naylor.H, Turner.P, Vaughan.D.J, Boyce.A.J, Fallick.A.E, 1989. Genetic studies of red bed mineralization in the Triassic of the Cheshire Basin, northwest England, *Journal of the Geological Society, London*, vol 146, pp.685-699

Newell. A.J., 2018. Rifts, rivers and climate recovery: A new model for the Triassic of England. *Proceedings of the Geologists' association*, vol., 129, pp., 352-371

Niedermayr.G, Beran.A, Branstätter.F, 1989. Diagenetic type magnesites in the Permo-Scythian rocks of the Eastern Alps, Austria. In: Möller.P (ed) *Magnesite Geology, Mineralogy, Geochemistry, Formation of Mg-Carbonates*, pp.35-59. Monograph Series on Mineral Deposits. Berlin, Stuttgart: Gebrüder Bornträger.

Nielsen, H., 1978. Sulfur isotopes in nature. In: Wedepohl.K.K (ed) *Handbook of Geochemistry*. Sect. 16-B. Springer, Berlin

Nielsen.H, Ricke.W, 1964. Schwefel-Isotopenverhältnisse von Evaporiten aus Deutschland; Ein Beitrag zur Kenntnis von $\delta^{34}S$ im Meerwasser-Sulfat, *Geochimica et Cosmochimica Acta*, vol 28, pp.577-591

Ogg, J.G., Ogg, G.M., Gradstein, F.M., 2016. *A concise geologic timescale 2016*. Amsterdam: Elsevier

Ortí.F, Guimerà..J, Götz.A.E, 2020. Middle-Upper Triassic stratigraphy and structure of the Alt Palància (eastern Iberian Chain): A multidisciplinary approach, *Geologica Acta*, vol. 18, pp.1-25

Ortí.F, Pérez-López.A, García-Veigas.J, Rosell.L, Cendón.D.I, Pérez-Valera.F, 2014. Sulfate isotope compositions ($\delta^{34}S$, $\delta^{18}O$) and strontium isotopic ratios ($^{87}Sr/^{86}Sr$) of Triassic evaporites in the Betic Cordillera (SE Spain), *Revista de la Sociedad Geológica de España*, vol 27(1), pp.79-89

Ortí, F., Pérez-López, A., Pérez-Valera, F., Benedicto, C., 2022. Isotope composition ($\delta^{34}S$, $\delta^{18}O$) of the Middle-Early Jurassic sulfates in eastern Iberia. *Sedimentary Geology*, p.106104

Ortí.F., Salvany.J.M., Rosell.L., Castelltort.X., Inglès.M., Playà.E., 2018. Middle Triassic evaporite sedimentation in the Catalan Basin: Implications for the paleogeographic evolution in the NE Iberian platform. *Sedimentary Geology*, vol. 374, pp.158-178

Owens, J.D., Gill, B.C., Jenkyns, H.C., Bates, S.M., Severmann, S., Kuypers, M.M.M., Woodfine, R.G., Lyons, T.M., 2013. Sulfur isotopes track the global extent and dynamics of euxinia during Cretaceous Oceanic Anoxic Event 2. *Proceedings of the National Academy of Sciences*, vol., 110(46), pp., 18407-18412

Pak.E, 1978. Schwefelisotopenuntersuchungen am Institut für Radiumforschung und Kernphysik II, *Anzeiger der Akademie der Wissenschaften Mathematisch-Naturwissenschaftliche Klasse*, pp.6-22

Pak.E, Schauberger.O, 1981. Die geologische Datierung der ostalpinen Salzlagerstätten mittels Schwefelisotopenuntersuchungen, *Verhandlungen der Geologischen Bundesanstalt*, pp.185-192

Pankina.R.G, Maksimov.S.P, Kalinko.M.K, Monakhov.I.B, Guriyeva.S.M, 1975. Sulfur isotopic composition in the Phanerozoic evaporites of Bulgaria, *Geochemistry International*, vol 12(6), pp.79-83

Parrish, J.T., 1993. Climate of the supercontinent Pangea. *The Journal of Geology*, vol., 101(2), pp., 215-233

Pasquier, V., Bryant, R.N., Fike, D.A., Halevy, I., 2021. Strong local, not global, controls on pyrite sulfur isotopes. *Science Advances*, vol., 7, pp., eabb7403

Pasqiuer, V., Sansjofre, P., Rabineau, M., Revillon, S., Houghton, J., Fike, D.A., 2017. Pyrite sulfur isotopes reveal glacial-interglacial environmental changes. *Proceedings of the National Academy of Sciences*, vol., 114, pp., 5941-5945

Paytan, A., Gray, E.T., Ma, Z., Erhardt, A., Faul, K., 2012. Application of sulphur isotopes for stratigraphic correlation. *Isotopes in Environmental and Health Studies*, vol., 48(1), pp., 195-206

Peacock, D.C.P., 2004. The post-Variscan development of the British Isles within a regional transfer zone influenced by orogenesis. *Journal of Structural Geology*, vol., 26(12), pp., 2225-2231

Pérez-López, A., Benedicto, C., Ortí, F., 2021. Middle Triassic carbonates of Eastern Iberia (Western Tethyan Realm): A shallow platform model. *Sedimentary Geology*, vol., 420, pp., 105904

Peryt.T.M, Halas.S, Hryniv.S.P, 2010. Sulphur and oxygen isotope signatures of late Permian Zechstein anhydrites, West Poland: seawater evolution and diagenetic constraints, *Geological Quarterly*, vol 54(4), pp.387-400

Posey.H.H, Fisher.R.S, 1989. A sulfur and strontium isotopic investigation of Lower Permian anhydrite, Palo Duro Basin, Texas, U.S.A., *Applied Geochemistry*, vol 4, pp.395-407

Present, T.M., Adkins, J.F., Fischer, W.W., 2020. Variability in sulfur isotope records of Phanerozoic seawater sulfate, *Geophysical Research Letters*, vol., 47(18), pp., 1-17

Qing.H, Nickel.E, Marsh.A, Gerla.G, Yang.C, 2005. Geochemistry of anhydrites at the IEA Weyburn site: implications for assessing the effectiveness of sealing rocks, *Greenhouse Gas Control Technologies*, vol 2, pp.1981-1982

Raab, M., Spiro, B., 1991. Sulfur isotopic variations during seawater evaporation with fractional crystallization, *Chemical Geology*, vol., 86, pp., 323-333

Raven, M.R., Fike, D.A., Bradley, A.S., Gomes, M.L., Owens, J.D., Webb, S.A., 2019. Paired organic matter and pyrite $\delta^{34}S$ records reveal mechanisms of carbon, sulfur, and iron cycle disruption during Ocean Anoxic Event 2. *Earth and Planetary Science Letters*, vol., 512, pp., 27-38

Rennie, V.C.F., Paris, G., Sessions, A.L., Abramovich, S., Turchyn, A.V., Adkins, J.F., 2018. Cenozoic record of $\delta^{34}S$ in foraminiferal calcite implies an early Eocene shift to deep-ocean sulfide burial. *Nature Geoscience*, vol., 11, pp., 761-765

Richardson, J.A., Keating, C., Lepland, A., Hints, O., Bradley, A.S., Fike, D.A., 2019. Silurian records of carbon and sulphur cycling from Estonia: The importance of depositional environments on isotopic trends. *Earth and Planetary Science Letters*, vol., 512, pp., 71-82

Rick.B, 1990. Sulphur and oxygen isotopic evolution of Swiss Gipskeuper (Upper Triassic), *Chemical Geology: Isotope Geoscience Section*, vol 80(3), pp.243-250

Sarg.J.F, 1981. Petrology of the carbonate-evaporite facies transition of the Seven Rivers Formation (Guadalupian, Permian), southeast New Mexico, *Journal of Sedimentary Petrology*, vol 51(1), pp.73-95

Schobben, M., Stebbins, A., Algeo, T.J., Strauss, H., Leda, L., Haas, J., Struck, U., Korn, D., Korte, C., 2017. Volatile earliest Triassic sulphur cycle: A consequence of persistent low seawater sulfate concentrations and a high sulfur cycle turnover. *Palaeogeography, Palaeoclimatology, Palaeoecology*, vol., 468, pp., 74-85

Schreiber, B.C., Tabakh, M.E., 2000. Deposition and early alteration of evaporites. *Sedimentology*, vol., 47, pp., 215-238

Shen, L., Wang, L., Liu, C., Zhao, Y., 2021. Sr, S, and O isotope compositions of evaporites in the Lanping-Simao Basin, China. *Minerals*, vol., 11(2), pp., 1-20

Smith, D.B., 1989. The late Permian palaeogeography of north-east England. *Proceedings of the Yorkshire Geological Society*, vol., 47(4), pp., 285-312

Solomon.M, Rafter.T.A, Dunham.K.C, 1971. Sulphur and oxygen isotope studies in the northern Pannines in relation to ore genesis, *Transactions of the Institution of Mining and Metallurgy Section B, 80B*, pp.259-275

Spötl.C, Pak.E, 1996. A strontium and sulfur isotopic study of Permo-Triassic evaporites in the Northern Calcareous Alps, Austria, *Chemical Geology*, vol 131, pp.219-234

Stebbins, A., Algeo, T.J., Olsen, C., Sano, H., Rowe, H., Hannigan, R., 2019. Sulfur-isotope evidence for recovery of seawater sulfate concentrations from a PTB minimum by the Smithian-Spathian transition. *Earth-Science Reviews*, vol., 195, pp., 83-95

Stemmerik.L, Piasecki.S, 2004. Isotopic evidence for the age of the Røde Ø Conglomerate, inner Scoresby Sund, East Greenland, *Bulletin of the Geological Society of Denmark*, vol 51, pp.137-140

Stemmerik.L, Rouse.J.E, Spiro.B, 1988. S-isotope studies of shallow water, laminated gypsum and associated evaporites, Upper Permian, East Greenland, *Sedimentary Geology*, vol 58, pp.37-46

Strehl.V.E, Niedermayr.G, Scheriau-Niedermayr.E, Pak.E, 1980. Die Gipsvorkommen an der Südseite des Dobratsch (Villacher Alpe), Kärnten, *Carinthia II*, pp.77-89

Sun, H., Xiao, Y., Gao, Y., Zhang, G., Casey, J.F., Xhen, Y., 2018. Rapid enhancement of chemical weathering recorded by extreme light seawater lithium isotopes at the Permian-Triassic boundary. *Proceedings of the National Academy of Sciences*, vol., 115(15), pp., 3782-3787

Thode, H.G., Monster, J., 1965. Sulfur-isotope geochemistry of petroleum, evaporites, and ancient seas. In: Young, A., Galley, J.E. (ed) *Fluids in subsurface environments AAPG Memoir 4*, pp., 367-377. Tulsa, OK: American Association of Petroleum Geologists.

Thode.H.G, Monster.J, 1970. Sulfur isotope abundances and genetic relations of oil accumulations in the Middle East Basin, *AAPG Bulletin*, vol 54(4), pp.627-637

Tucker, M.E., 1991. Sequence stratigraphy of carbonate-evaporite basins: models and applications to the Upper Permian (Zechstein) of northeast England and adjoining North Sea. *Journal of the Geological Society*, London, vol., 148, pp., 1019-1036

Van Driessche, A.E.S., Canals, A., Ossorio, M., Reyes, R.C., García-Ruiz, J.M., 2016. Unraveling the sulfate sources of (giant) gypsum crystals using gypsum isotope fractionation factors, *The Journal of Geology*, vol., 124, pp., 235-245

Vredenburgh.L.D, Cheney.E.S, 1971. Sulfur and carbon isotopic investigation of petroleum, Wind River Basin, Wyoming, *AAPG Bulletin*, vol 55(11), pp.1954-1975

Warren, J.K., 2010. Evaporites through time: Tectonic, climatic and eustatic controls in marine and nonmarine deposits. *Earth-Science Reviews*, vol., 93(3-4), pp., 217-268

Warrington, G., Ivimey-Cook, H.C., 1992. Triassic. In: Cope, J.C.W., Ingham, J.K., Rawson, P.F., (eds) *Atlas of palaeogeography and lithofacies*. Geological Society, London, Memoirs, vol., 13(1), pp., 97-106

Werne, J.P., Lyons, T.W., Hallander, D.J., Formolo, M.J., Sinninghe Damsté, J.S., 2003. Reduced sulfur in euxinic sediments of the Cariaco Basin: sulfur isotope constraints on organic sufur formation. *Chemical Geology*, vol., 195, pp., 159-179

Wignall, P.B., Bond, D.P.G., 2008. The end-Triassic and Early Jurassic mass extinction records in the British Isles. *Proceedings of the Geologists' Association*, vol., 119, pp., 73-84

Woods, P.J.E., 1973. Potash exploration in Yorkshire: Boulby Mine pilot borehole. *Transactions (Section B) Institution of Mining and Metallurgy*, vol., 82, pp., 99-106

Worden, R.H., Smalley, P.C., Fallick, A.E., 1997. Sulfur cycle in buried evaporites. *Geology*, vol., 25, pp., 643-646

Yeremenko, N.A., Pankina, R.G., 1972. Variations of $\delta^{34}S$ in sulfates of recent and ancient marine basins of the Soviet Union. *Geochemistry International*, vol., 10, pp., 45-54
